# Anisotropic diffraction of materials with fibre symmetry: application to chitin cuticle

**DOI:** 10.1107/S2052252525009686

**Published:** 2026-01-01

**Authors:** Yanhong Wang, Tim Snow, Nick Terrill, Himadri Shikhar Gupta

**Affiliations:** a School of Engineering and Materials Science Queen Mary University of London Mile End Road LondonE1 4NS United Kingdom; b Diamond Light Source Harwell United Kingdom; UCL, United Kingdom

**Keywords:** composite materials, materials modelling, nanostructure, imaging, materials science, wide-angle X-ray diffraction, WAXD, fibres

## Abstract

Understanding the structure–function relationships in anisotropic fibre-symmetric materials is critical for both biological insight and bioinspired design. We present a generalized analytical model for X-ray diffraction intensity from nanofibrillar materials with fibre symmetry, accommodating arbitrary diffraction rings beyond prior axial and equatorial limits. This model integrates 3D orientation, strain heterogeneity and angular misalignment effects, and is validated using wide-angle X-ray diffraction (WAXD) from the Bouligand-structured cuticle of the mantis shrimp (*Odontodactylus scyllarus*). Using scanning synchrotron WAXD, we extract depth-averaged and sub-lamellar information on 3D fibre orientation and crystalline parameters from 2D scans. Model simulations and experimental fits show accurate reconstruction of the Bouligand texture and reveal spatial gradients in orientation, strain and angular dispersion. By fitting multiple reflections – axial (002), equatorial (110) and intermediate (013) – we improve the robustness in parameter extraction, especially in regions where the Ewald condition is partially satisfied. Our framework enhances the interpretation of WAXD in heterogeneous fibre-based materials and can be embedded into advanced tomographic or machine-learning workflows. This approach is applicable to a broad class of biological and synthetic composites, facilitating high-throughput structural characterization in scenarios where rotation is impractical or impossible.

## Abstract 2

A generalized diffraction model is presented for anisotropic nanomaterials with fibre symmetry, enabling the extraction of 3D orientation, strain and angular dispersion from wide-angle X-ray scattering patterns without sample rotation. Validated on chitinous Bouligand structures in shrimp cuticle, this framework allows robust multi-reflection analysis and is broadly applicable to complex biological and synthetic fibre-based materials.

## 1. Introduction and background

The functional properties of many materials are closely linked to their structural arrangement across multiple length scales. This is especially evident in various biological systems, where the structural building blocks exhibit a hierarchical organization spanning nanoscale to macroscale dimensions (Eder *et al.*, 2018 ▸; Bechthold & Weaver, 2017 ▸; Grünewald *et al.*, 2024 ▸). Such hierarchical structuring is common in nature and has inspired advances in materials science, offering an efficient strategy to achieve desired functionalities with minimal weight and low cost (Nepal *et al.*, 2023 ▸; Chen *et al.*, 2020 ▸; Jia *et al.*, 2021 ▸). Some prototypical examples include chitin fibres, silk fibroin, cellulose and peptide-based fibres (Seidl *et al.*, 2021 ▸; Weaver *et al.*, 2012 ▸; Nishiyama, 2009 ▸; Kim *et al.*, 2003 ▸; Woolfson & Ryadnov, 2006 ▸; Heinze, 2015 ▸; Marsh *et al.*, 1955 ▸). Therefore, investigating nanostructures across macroscopic scales is important for understanding the mechanical and functional properties of hierarchically organized systems. This will be helpful in exploring structure–property–function relationships in biological materials and in designing innovative bioinspired functional materials.

X-ray imaging techniques have been extensively applied to studies of hierarchical materials, as X-rays have a high penetration depth in dense materials such as mineralized bone (Almer & Stock, 2007 ▸; Gupta *et al.*, 2006 ▸; Liebi *et al.*, 2015 ▸) and arthropod cuticle (Al-Sawalmih *et al.*, 2008 ▸; Dong *et al.*, 2022 ▸; Sviben *et al.*, 2020 ▸). Small-angle X-ray scattering (SAXS) and wide-angle X-ray diffraction (WAXD) enable structural analysis ranging from molecular to atomic length scales, respectively, including the highly anisotropic orientation of collagen fibres (Kinney *et al.*, 2001 ▸; Moger *et al.*, 2007 ▸; Silva Barreto *et al.*, 2024 ▸). Furthermore, raster scanning has been reported useful in studying the mechanical response and the impact of structural heterogeneity on material mechanics (Silva Barreto *et al.*, 2023 ▸; Badar *et al.*, 2025 ▸). Diffraction and tensor tomography further extend these capabilities by incorporating sample rotation, enabling three-dimensional (3D) reconstructions of material structures combining two-dimensional (2D) raster scans with rotations around multiple axes, mainly reporting orientation of the nanocrystalline units but also wavevector resolved crystalline parameters (Palle *et al.*, 2020 ▸; Grünewald *et al.*, 2020 ▸; Liebi *et al.*, 2018 ▸; Schaff *et al.*, 2015 ▸; Zhao *et al.*, 2024 ▸). On the X-ray diffraction tomography side, advances include the development of rapid texture tomography techniques to reconstruct the orientation distribution function and use of Laue energy-dispersive diffraction methods, *e.g.* on tooth (Frewein *et al.*, 2024 ▸; Carlsen *et al.*, 2025 ▸; Sakr *et al.*, 2024 ▸).

In many materials applications, however, carrying out tomographic rotation may not always be practical or desired, but some level of 3D ultrastructural information is still desirable to interpret reflection peak shifts or intensity changes appropriately. Examples include potential radiation damage during high-throughput scanning of tissue sections, limitations in kinetic measurements during load application, or situations where multi-axial rotation is not practically available. In this regard, many biological materials like bone, cuticle and tendon exhibit a form of fibre symmetry at the supramolecular scale, which facilitates 3D information extraction without sample rotation. Exploiting this, the principal fibre direction of cellulose fibrils was extracted by modelling the equatorial diffraction spots as rings in pioneering work over two decades ago (Lichtenegger *et al.*, 1999 ▸; Lichtenegger *et al.*, 2003 ▸; Paris & Müller, 2003 ▸; Ogurreck *et al.*, 2010 ▸, 2013 ▸). The formulation was extended by our group to model the axial and equatorial diffraction rings of chitin fibrils in the natural biogenic armours of stomatopod cuticle, including their spatial orientation gradient (Zhang *et al.*, 2016 ▸), fibrillar deformation (Zhang *et al.*, 2017 ▸) and pre-strain changes under compression (Zhang *et al.*, 2020 ▸). In parallel, Ogurreck *et al.* (2010 ▸, 2013 ▸) reported analyses which predicted the positions of diffraction spots for the arbitrary (*hkl*) diffraction peaks from cellulose nanocrystals in wood cell walls. More recently, Raviv and co-workers have extended the *D+* solution scattering software package developed in their group (Ginsburg *et al.*, 2019 ▸) to predict 2D scattering from *e.g.* fibres oriented in a specific direction (although at supra­molecular levels) (Balken *et al.*, 2023 ▸). Nevertheless, the general diffraction intensity expression, peak locations and widths for an arbitrary diffraction ring (axial, equatorial or in between) and their aggregation into planar fibrillar arrays like the Bouligand structure have not, to the best of our knowledge, been reported. We present this model here and validate it by applying it – at two different micro- and nanofocus X-ray resolution levels – to the ultrastructure of stomatopod tergite cuticle. Tergite cuticle is used as a test case because the Bouligand plywood structure of the chitin fibrils (a spiralling rotation) is well established as ground truth from other work (Zimmermann *et al.*, 2013 ▸). This model applies not only to chitin-based materials, but also to a wide range of nano­structured anisotropic materials with fibre symmetry, and can be integrated with tomographic methods.

We first describe the experimental measurements to acquire the scanning X-ray diffraction measurements on cuticle, followed by a mathematical description of the model and some of its characteristics, and the application of the model to the cuticle diffraction data.

## 2. Materials and methods

### 2.1. Sample preparation

**Figure 1:**
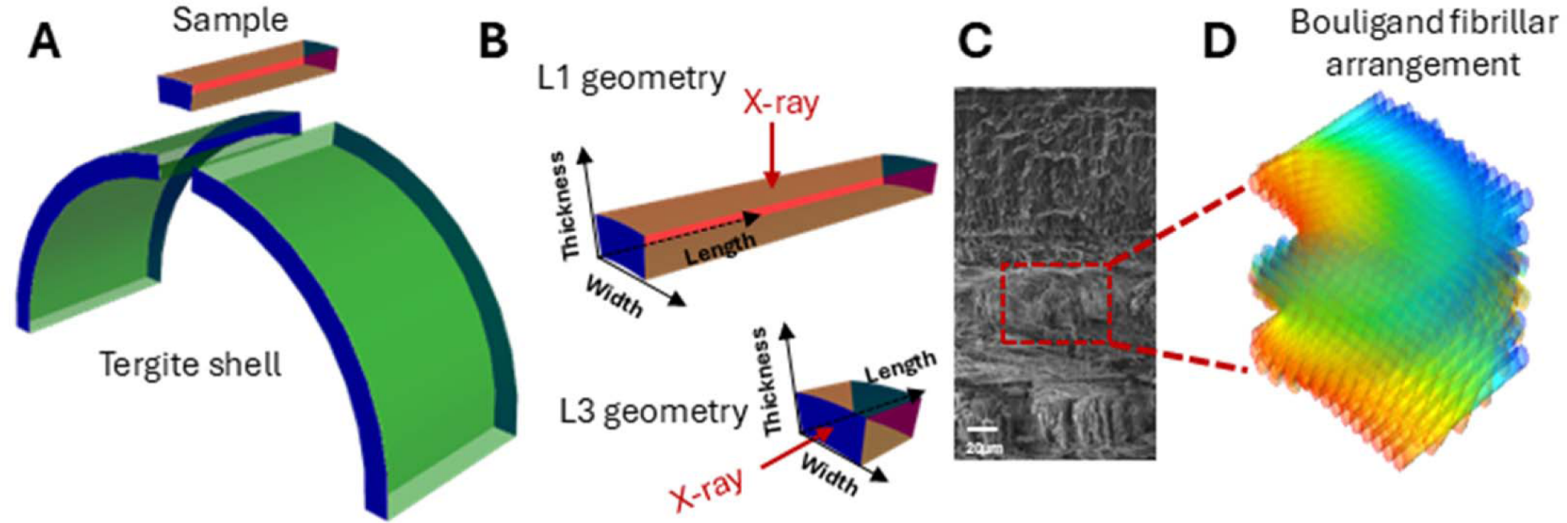
Cuticle sample geometry and structure. (*a*) Schematic diagram of a tergite shell from mantis shrimp and the strip samples used in the experiments. (*b*) Cuticle samples with different geometries. L1 geometry is when the cuticle surface is perpendicular to the X-ray beam and L3 geometry is when the cuticle surface is parallel to the X-ray beam. The dimensions were roughly 3 mm (length), 0.5 mm (width) and 0.3 mm (thickness) for L1-oriented samples; and around 0.5 mm (length), 0.5 mm (width) and 0.3 mm (thickness) for L3-oriented samples. (*c*) SEM image showing the cuticle microstructure with fibres of different orientations. (*d*) Schematic diagram showing the Bouligand fibrillar structure.

Adult mantis shrimp (*Odontodactylus scyllarus*) were supplied by a local supplier (Tropical Marine Centre, London) and stored at −20°C until used for sample preparation. The cuticle samples (Fig. 1 ▸) were prepared following established protocols we have previously reported (Zhang *et al.*, 2016 ▸; Zhang *et al.*, 2017 ▸; Zhang *et al.*, 2020 ▸; Wang *et al.*, 2019 ▸). Briefly, the abdomen tergite cuticle [Fig. 1 ▸(*a*)] was carefully dissected and strips were sectioned along the axis of the animal; the dimensions of the strip samples were ∼3 × 0.5 × 0.3 mm (length × width × thickness). These specimens were then stored at −20°C until used for experiments.

### 2.2. X-ray diffraction data collection

The cuticle samples were hydrated in water after removal from the freezer and then mounted on sample holders. The samples were exposed to ambient air without additional humidity during the X-ray measurements.

For the measurement on cuticle samples of L1 geometry [Fig. 1 ▸(*b*)], data were collected on beamline I22 at Diamond Light Source (DLS, Harwell, UK) (Smith *et al.*, 2021 ▸). Three cuticle samples were tested (*n* = 3, labelled S01, S02 and S03) and their dimensions were ∼3 × 0.5 × 0.3 mm (length × width × thickness). An X-ray beam of 14 keV was applied perpendicular to the cuticle surface, with a beam size of 15 × 15 µm at the sample. Individual WAXD patterns (2–5 frames per sample) were taken from selected points in the middle area of the samples. X-ray data were captured using a Pilatus P3-2M detector (Kraft *et al.*, 2009 ▸), which is composed of 3 (with 7-pixel gaps) × 8 (with 17-pixel gaps) modules, with 1475 × 1679 pixels and 172 × 172 µm pixel size. WAXD patterns were recorded at a sample-to-detector distance of 296 mm, with a 1 s exposure per frame.

To record the WAXD patterns from single fibres, X-ray data were collected from the cross-sectional area of a single cuticle sample [L3 geometry, Fig. 1 ▸(*b*)] on beamline P06 at PETRA III, DESY (Hamburg, Germany) (Schroer *et al.*, 2010 ▸). The dimensions of the sample were ∼0.5 × 0.5 × 0.3 (mm, length × width × thickness). A monochromatic X-ray beam (17 keV) with a beam size of 7 (horizontal) × 3 µm (vertical) was applied parallel to the cuticle surface and a map scan was performed in the middle of the cross-sectional area, with a scan area of ∼150 × 300 µm (along the cuticle width and thickness directions, respectively). A total of 2121 frames were collected, with 21 columns along the width direction with a 7 µm step and 101 rows along the thickness with a 3 µm step. The exposure time was 1 s per frame and the sample-to-detector distance was 166 mm. WAXD data were collected on an Eiger 4M detector (Dinapoli *et al.*, 2013 ▸), consisting of eight modules with 37-pixel gaps dividing the vertical axis into four sections and 10-pixel gaps dividing the horizontal axis into two sections, with a resolution of 2070 × 2167 pixels and 75 × 75 µm pixel size.

### 2.3. X-ray diffraction data analysis

The two-dimensional WAXD patterns were analysed using* Fit2D* (Hammersley, 2016 ▸) and the Python packages *lmfit* (Newville *et al.*, 2025 ▸) and *matplotlib* (Hunter, 2007 ▸) in an Anaconda environment. Azimuthal intensity profiles *I*_*hkl*_(χ), where (*hkl*) refers to the (002), (110) and (013) chitin reflections, were integrated over the following radial regions: 11.8–12.8 nm^−1^ for (002), 12.8–15.5 nm^−1^ for (110) and 18.0–19.2 nm^−1^ for (013), across the full azimuthal range (χ, 0–360°, measured from the horizontal axis). A ring background subtraction method was used to remove the diffuse scattering background, as previously described (Zhang *et al.*, 2017 ▸). We averaged the intensity from two rings with a radial width of 0.2 nm^−1^ – an inner ring and an outer ring close to the specific reflection – as background and removed it from the *I*_*hkl*_(χ) profiles. Note that for the (002) reflection in L3 geometry, only an inner ring was subtracted, due to the broad and diffuse (110) reflection, which made it impossible to remove an outer ring between the (002) and (110) reflections. Before background subtraction, data points within the detector module gaps (with intensities decreasing to nearly zero) were removed from each ring, leading to missing points in the profiles. Radial intensity profiles *I*_*hkl*_(*q*) were integrated over the range of 4.1–25.7 nm^−1^ and multiple reflections were fitted using Gaussian functions. From the fits, the lattice spacing [*d*_(*hkl*)_ = 2π/*q*_(*hkl*)_, where *q*_(*hkl*)_ is the radial peak position], the width of the radial peak [σ_(*hkl*)_] and the radial intensity (the area under the radial profile) of each reflection were calculated.

## 3. Results and modelling

In previous studies, we focused on the axial and equatorial reflections, *i.e.* the (002) and (110) reflections from chitin fibres, respectively (Zhang *et al.*, 2017 ▸; Zhang *et al.*, 2020 ▸; Wang *et al.*, 2019 ▸). These two reflections were used to quantify the axial and radial fibrillar crystalline deformations, and a model for the (110) reflection was developed to determine the orientation of the chitin fibres. Here we extend our model to more general intermediate (*hkl*) reflections — specifically, the (013) reflection — to broaden its applicability. Below we present this model at multiple conditions: from a single fibre to a fibre array with a uniform orientation and then to fibres exhibiting a non-zero orientation distribution.

### 3.1. Diffraction from fibre-symmetric materials: single fibril and array of fibrils

**Figure 2:**
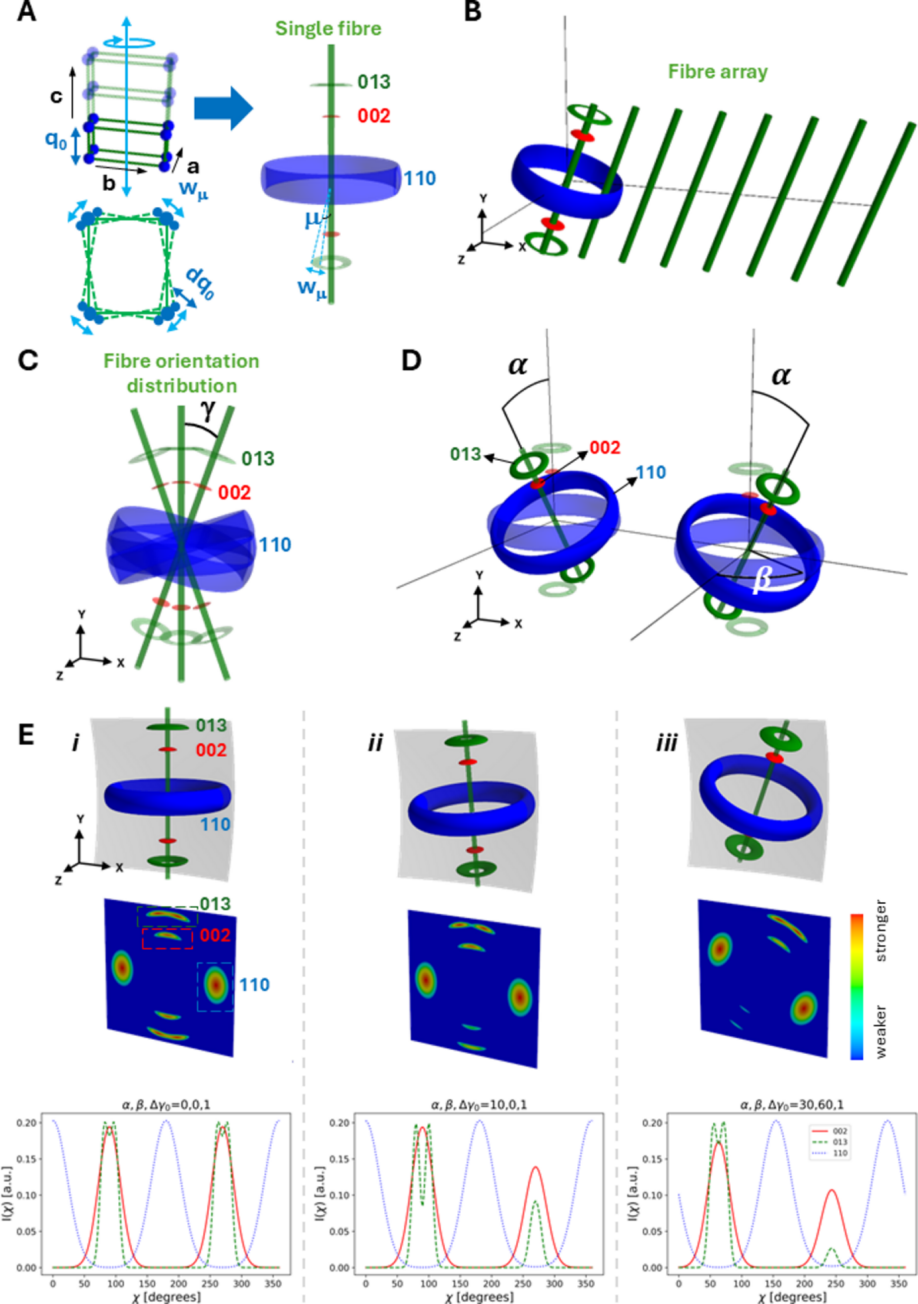
The α-chitin structure and X-ray diffraction model for a single chitin fibre and a fibre array at different tilt conditions. (*a*) (Left, top) the ortho­rhombic structure of α-chitin. The three directions are labelled *a*, *b* and *c*, with the *c* axis along the fibril axis. (*a*) (Left, bottom) Small variations in the unit-cell orientation lead to angular broadening *w*_μ_ and radial broadening *dq*_0_. (*a*) (Right) A single chitin fibril (green cylinder) and the reciprocal-lattice reflections distributed as symmetric rings around the *c* axis due to fibre symmetry (red 002, blue 110, green 013); μ is the opening angle between reflection rings and the *c* axis. (*b*) An array of chitin fibrils (green cylinders) with the same orientation and the three generated prototypical reciprocal-lattice intensity bands. The same colours are used for the three reflection rings as in panel (*a*). (*c*) A chitin fibril array with different orientations and the diffraction distribution in reciprocal space. γ is the distribution angle of fibres in the *x*–*y* plane. (*d*) A single chitin fibre (green cylinder) with (left) a tilt angle of α and (right) tilt angles of (α, β). The translucent diffraction rings are from a single chitin fibre without tilt (α = 0°, β = 0°). (*e*) (Top row) Diffraction rings in the fibre frame coordinates, (middle row) 2D X-ray patterns in laboratory coordinates and (bottom row) 1D intensity distributions along the azimuthal direction (χ) at different tilt conditions. The chitin fibre distribution shown here is small, with Δγ_0_ = 1°. Column (*i*) no tilt, α = 0°, β = 0°; column (*ii*) small α tilt, α = 10°, β = 0°; column (*iii*) large tilt angles, α = 30°, β = 60°. The same colours are used in the 1D profiles of the three reflections as for the diffraction rings in reciprocal space [panel (*a*)].

Consider a 3D reciprocal-space coordinate system {**Q**^F^} which is aligned with the fibre (fibre is used interchangeably with fibril in the current work), with the *y* axis being parallel to the fibre axis. We will derive expressions for the reciprocal-space intensity in this fibre-fixed coordinate before transforming to the laboratory coordinate system {**Q**}. For the orthorhombic structure of chitin [Fig. 2 ▸(*a*)], due to fibre symmetry in the scattering volume around the *c* axis, the reciprocal-space node at reciprocal-lattice vector (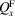, 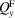, 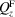) = [*h*(2π/*a*), *k*(2π/*b*), *l*(2π/*c*)] [the (*hkl*) reflection] gets distributed into a ring around the *c* axis, as shown schematically in Figs. 2 ▸(*a*) and 2 ▸(*b*) [for the green rings (*hkl*) with *k* or *l*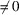 and blue rings (*hk*0) only]. The opening angle between the reflection ring and the *c* axis is denoted μ, which is 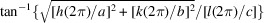 for the (*hkl*) reflection and 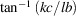 for the (0*kl*) reflection. A symmetric ring is located at π − μ for the (0*kl*) reflection [*e.g.* the (002) and (013) reflections in Fig. 2 ▸(*a*)].

We model the intensity of these rings of a general (*hkl*) reflection as the product of two orthogonal peak intensity profiles in reciprocal space, one along the 3D radial *q* axis centred at *q*_0_ (the centre of the radius *q* range where a reflection occurs) and the second on the unit sphere. The reciprocal-space intensity for an (*hkl*) reflection, with angle μ, from a single fibril 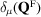 is
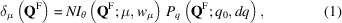
where *I*_θ_ and *P*_*q*_ denote the intensity profiles on the unit sphere and along the radial axis, respectively, and *N* is a constant depending on the material, structure factor and detector. Here, we take the intensity profile *I*_θ_ on the unit sphere to consist of two symmetric bands with a Lorentzian shape, as a function of the polar angle from the fibre axis and centred at μ and π − μ (for the ±*c* directions). If we define 
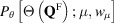
 as a Lorentzian peak function with argument 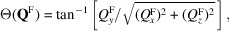
centre π/2 − μ and angular width parameter *w*_μ_, it can be seen that in *q* space this function shows this symmetric band behaviour (Fig. S1 in the supporting information). The argument 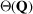 is the angle between the vector **Q** and the *Q_x_–Q_z_* plane; the (*hkl*) ring is at an angle π/2 − μ to this plane and hence, to maximize the intensity, the centre of the peak *P*_θ_ is at π/2 − μ. The radial peak profile *P*_*q*_ is taken as a Gaussian with centre *q*_0_ and radial width *dq*.

Due to misalignment between the crystallites, angular broadening (increasing *w*_μ_) can occur and some reflections [*e.g.* the equatorial (110)] are much wider than *e.g.* the axial (002) reflection. As well established, mechanical stresses on such nanocrystallite assemblies can give rise to both strain broadening and shifts in peak position. The choice of a Lorentzian (versus a Gaussian) profile for *P*_θ_ is due to a better match with the experimental data. As a result, the function *P*_θ_ will have peaks at the locations of maximum intensity on the 3D sphere: polar angle θ (with respect to *Q*_*y*_) of 0 for (002), π/2 for (110) and intermediate for (013) [see also Fig. S1(*b*)]. However, rotation (both in and out of the plane) will change this behaviour, as described below.

If the fibre is rotated in the *x*–*y* plane by γ (as occurs when it is part of a planar array of fibrils like the Bouligand layers in cuticle) as shown in Fig. 2 ▸(*c*), the argument 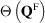 is
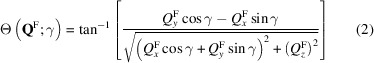
and the intensity of the tilted fibril is
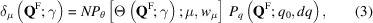
where 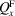, 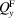 and 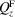 are the vectors along the *x*, *y* and *z* directions, respectively, in the fibre coordinates. For a planar array of fibrils rotated in the *x*–*y* plane, the total diffraction intensity is an integral over the angular distribution function *w*(γ; {γ_0_, Δγ_0_}) [equation (S1) in the supporting information].

### 3.2. Effects of 3D tilted fibril arrays on 2D patterns

If the fibril array is tilted in three dimensions out of the *x*–*y* plane in the laboratory frame by the angles (α, β) as shown in Fig. 2 ▸(*d*), the measured detector intensity requires first transforming the reflection intensity from fibre coordinate reciprocal wavevectors **Q**^F^ to laboratory coordinate reciprocal wavevectors **Q** and then the Ewald diffraction condition applied to consider **Q** = **q**, where the scattering vector **q** has magnitude 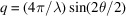 (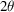 being the scattering angle). Like our prior work (Zhang *et al.*, 2016 ▸; Zhang *et al.*, 2017 ▸; Zhang *et al.*, 2020 ▸), for the reciprocal-space intensity for an (*hkl*) reflection 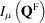, integrating over all individual fibrils with all distributions and rotations, we have (in the fibre frame)
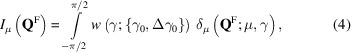
where 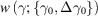 is the angular distribution function, γ is the rotation angle of fibrils in the *x*–*y* plane, 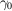 and 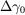 are the centre and width parameters, respectively, of the fibril distribution, 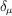 is the reciprocal-space intensity from a single fibril as defined earlier and expressed in equations (1) and (3), 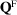 is the reciprocal-space wavevector in the fibre frame, and μ is the angle between the reflection rings and the *c* axis of the chitin crystal as defined earlier. The equivalent expression in laboratory-based reciprocal-space vectors **Q** can be obtained by applying rotation matrices to the fibre-frame reciprocal-space vectors **Q**^F^ and then restricting to wavevectors **Q** = **q** satisfying the Ewald condition, as described by us (Zhang *et al.*, 2016 ▸; Zhang *et al.*, 2017 ▸) and others [*e.g.* Frewein *et al.* (2024 ▸)].

Fig. 2 ▸(*e*) shows, for nonzero tilts of the principal fibre direction, how the real-space and 3D reciprocal-space structures vary and the consequent effects on the 2D X-ray patterns and 1D azimuthal intensity profiles. For graphical simplicity, the fibre is shown along the principal direction in the top row, but the simulated structure leading to the WAXD pattern is derived from a more realistic Gaussian distribution of fibres around the same principal direction in the *x*–*y* plane [unrotated case, Fig. 2 ▸(e-*i*)]. We see that asymmetric peak intensity changes occur for α-tilts [out of plane, Fig. 2 ▸(e-*ii*)], and when combined with β-tilts [Fig. 2 ▸(e-*iii*)] both intensity changes and rotation on the detector plane are visible. The details of the azimuthal intensity formula and how the general equation (4) reduces to the limiting cases of axial (00*l*) and equatorial (*hk*0) are shown in the supporting information (Zhang *et al.*, 2016 ▸; Zhang *et al.*, 2017 ▸).

### 3.3. General 2D diffracted intensity

For reciprocal-space vectors satisfying the diffraction condition, using the relation between fibre (**Q**^F^) and laboratory reciprocal-space coordinates (**Q**) and applying the Ewald diffraction condition, the expression for 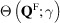 [equation (2)] can be written in terms of the tilt angles (α, β, γ), the scattering vector magnitude *q* and the azimuthal angle χ [supporting information, equation (S2)]. The vector **Q**^F^ in the fibre frame is
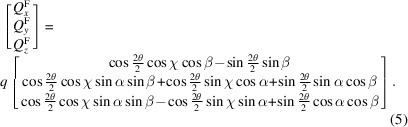
Applying the above expression into the relation for 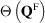 and 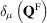 leads to the final expression for the azimuthal diffraction intensity, obtained by fixing *q* = *q*_0_.

Complete characterization of the fibrous array requires extracting the orientation (α, β, γ_0_), the strain heterogeneity *dq*_0_, and the angular dispersion at the fibril (*w*_μ_) and fibre-array level (Δγ_0_). For this, a fit to the full 2D or integrated 1D pattern is necessary. However, as shown earlier for cellulose fibrils using similar principles (Lichtenegger *et al.*, 1999 ▸; Lichtenegger *et al.*, 2003 ▸) for the (110) reflection, the characteristics of the 2D pattern can be linked to the underlying model parameters for an initial estimate of the parameters and quicker fitting. Here we present results for the axial and equatorial tilt angles and the angular misalignment parameters for the single fibril case. The calculations leading to the tilt angles and the estimation of misalignment parameters like *w*_μ_ [in the same theme as earlier work (Stribeck, 2009 ▸; Polanyi, 2021 ▸) estimating fibre angles] are provided in the supporting information. Generalization to the planar array case is possible but will require numerical inversion.

**Figure 3:**
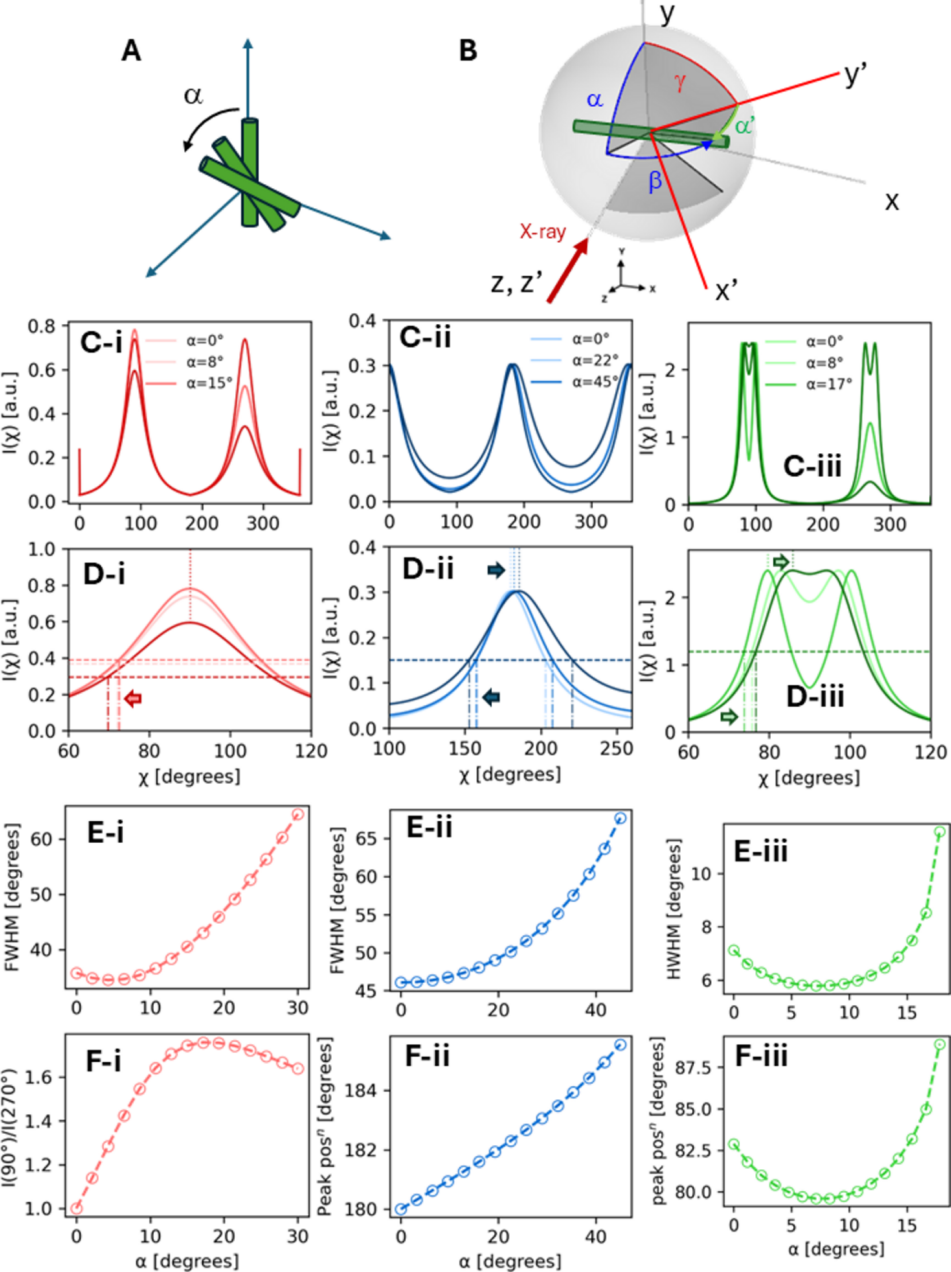
(*a*) Schematic diagram of chitin fibre rotated by varying tilt angle α. (*b*) Two equivalent ways of rotating a fibre to a specific 3D orientation: the (α, β) convention used for the model evaluation and the (γ, α′) option [equations (7) and (8)]. (c-*i*) to (c-*iii*) For (002) (red), (110) (blue) and (013) (green), *I*(χ) is plotted for increasing χ over 0–360°. (d-*i*) to (d-*iii*) Enlarged insets for each case around the main peaks below 180° azimuthal angle. A different α range is plotted in each case to show the changes more clearly. Vertical dotted lines from the top *x* axes indicate the azimuthal peak position [for (013), the left-hand split peak is taken as the peak position]. Vertical dot-dashed lines from the bottom *x* axes indicate the left-hand side of the angles at half-width at half-maximum (HWHM). Horizontal dashed lines indicate the half-maximum intensity. Horizontal block arrows are guides to the eye, indicating shifts of the peak centres, FWHM [full width at half-maximum, for the (002) and (110) reflections] or HWHM [for the (013) reflection]. (e-*i*) to (e-*iii*) Model predictions of changes in the FWHMs for the (002) and (110) reflections and in HWHM for the (013) reflection with varying tilt angle α. (f-*i*) Intensity ratio of the azimuthal peaks at 90° to 270° [*I*(90°)/*I*(270°)] for the (002) reflection with varying tilt angle α. (f-*ii*) and (f-*iii*) Azimuthal peak positions of the (110) reflection (at ∼180°) and the (013) reflection as indicated by the dotted vertical lines in panel (d-*iii*), plotted with varying tilt angle α. The relevant relations are given in equations (S8), (S11), (S13), (S15), (S18) and (S28) in the supporting information.

Assuming a single main fibre direction [*i.e.*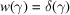], it is possible to estimate the tilt angles α, β and the width parameters *w*_μ_ from the peak positions, relative peak heights and peak widths, and peak separation. Since any 3D tilt can be expressed as either an (α, β) rotation or a (γ, α′) rotation [Figs. 3 ▸(*a*) and 3 ▸(*b*)], it is sufficient to consider an α rotation only. We orient ourselves such that the fibre is pointing vertically along *Q_y_*. The (00*l*), (*hk*0) and (*hkl*) reflections are considered separately (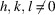). The expression for 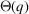 in 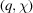 for nonzero α is
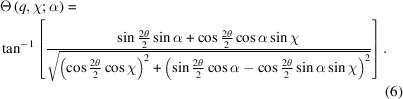


#### 3.3.1. (00*l*) reflection

The (00*l*) reflections including (002) are characterized by two peaks separated by 180° [Fig. 3 ▸(c-*i*)]. On fibre tilting in α, the principal reciprocal-space spherical sectors rotate out of the Ewald condition and the main changes are (i) a change in peak FWHM (full width at half maximum) for the two peaks [Figs. 3 ▸(c-*i*), 3 ▸(d-*i*) and 3 ▸(e-*i*)] and (ii) an increase in the upper (χ = 90°) to lower (χ = 270°) peak intensity ratios [Fig. 3 ▸(f-*i*)]. By finding the extrema for the term involving 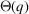 in the Lorentz intensity expression 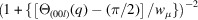, these are at π/2 and 3π/2 (as expected). From the above, the peak intensity ratios and FWHMs can be evaluated and are reported in Table 1 ▸ (detailed calculations are given in the supporting information). Figs. 3 ▸(c-*i*) and 3 ▸(d-*i*) show the change in the simulated *I*(χ) profiles of the (002) reflection with increasing tilt angle α. The predicted changes in FWHM and intensity ratio are plotted in Figs. 3 ▸(e-*i*) and 3 ▸(f-*i*) for a fixed *w*_μ_. We can use these relations to estimate α and *w*_μ_ by varying them until the peak intensity ratios and FWHMs match the experimental values (*e.g.* by constructing a look-up table).

#### 3.3.2. (*hk*0) reflection

In contrast to (00*l*), on tilting in α the equatorial (*hk*0) reflection stays at the same peak height but shifts slightly in angular χ position, because the reciprocal-space circular band of intensity is always intersected fully, except for very large tilt angles where it is sliced ‘through’ the band. The Ewald sphere curvature leads to a slight asymmetry in the peak separation, which can be less than or greater than 180° [Figs. 3 ▸(c-*ii*) and 3 ▸(d-*ii*)]. As for the (00*l*) case above, from the intensity expression for the (*hk*0) case, which is 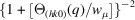, the azimuthal peak centres and FHWMs [Figs. 3 ▸(e-*ii*) and 3 ▸(f-*ii*)] can be extracted and are reported in Table 1 ▸. Tilt angles and *w*_μ_ can be estimated by simultaneously solving the equations or using a look-up table.

#### 3.3.3. (*hkl*) reflection

The characteristic double-ring form of (*hkl*) leads to two distinct regimes – for small α, a split-peak shape in at least one of the intensity maxima, and for 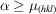 a rapidly decreasing single-peak form on either side [Figs. 3 ▸(c-*iii*) and 3 ▸(d-*iii*)]. A split-peak form indicates a fibre which is close to perpendicular to the X-ray beam (α = 0). For small α, the upper peaks (χ ≃ 90°) first diverge and then converge to 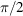, and a similar procedure to that used in the previous two cases, considering the intensity expression 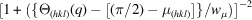, leads to expressions for the HWHMs (half widths at half maximum) and azimuthal peak positions shown in Figs. 3 ▸(e-*iii*) and 3 ▸(f-*iii*), and these parameters are also given in Table 1 ▸.

In general, the fibre rotates in both α and β. However, we can rotate the coordinate system around the beam direction (*i.e.* the *z* axis) by an angle γ such that the fibre projection in the rotated *x*–*y* plane is vertical. As sketched in Fig. 3 ▸(*b*), the fibre can be rotated by a second rotation angle α′ around the new *x* axis (*x*′) to reach the same 3D position as achieved with (α, β). It can be written as
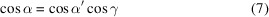
and
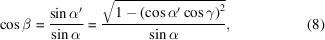
which can be solved for β. Thus, beyond the single α-tilt case above, combining equations (5), (6) and (7) enables an initial estimation of the tilt angles α′ (from Table 1 ▸ relations), from which α and β can be estimated using the relations for either (00*l*) for small tilts or for (*hkl*) for larger tilts.

## 4. Experimental validation

### 4.1. Microbeam with full lamellar averaging

As shown in Fig. 1 ▸(*b*), when the X-ray beam is normal to the cuticle surface (L1 geometry), it passes through all the cuticle sublamellae. To model this, we take the average of the signal from a full Bouligand stack, *i.e. w*(γ) = 1/π in equation (4), and fit the azimuthally integrated profiles of the (002), (013) and (110) reflections for μ = 0, μ_(013)_ and π/2, respectively. To fit the intensity *I*(χ) for each reflection and get the fibril orientation, a fit with shared parameters was implemented using the nonlinear minimization package *lmfit* (Nelder–Mead algorithm) in Python, with the model defined in previous sections. Thus the tilt angles (α, β) were shared as common fitting parameters across the *I*(χ) data across three reflections. Unit-cell estimates of the orthorhombic chitin structure were taken from Al-Sawalmih *et al.* (2008 ▸). The detailed fitting process is given in the supporting information.

**Figure 4:**
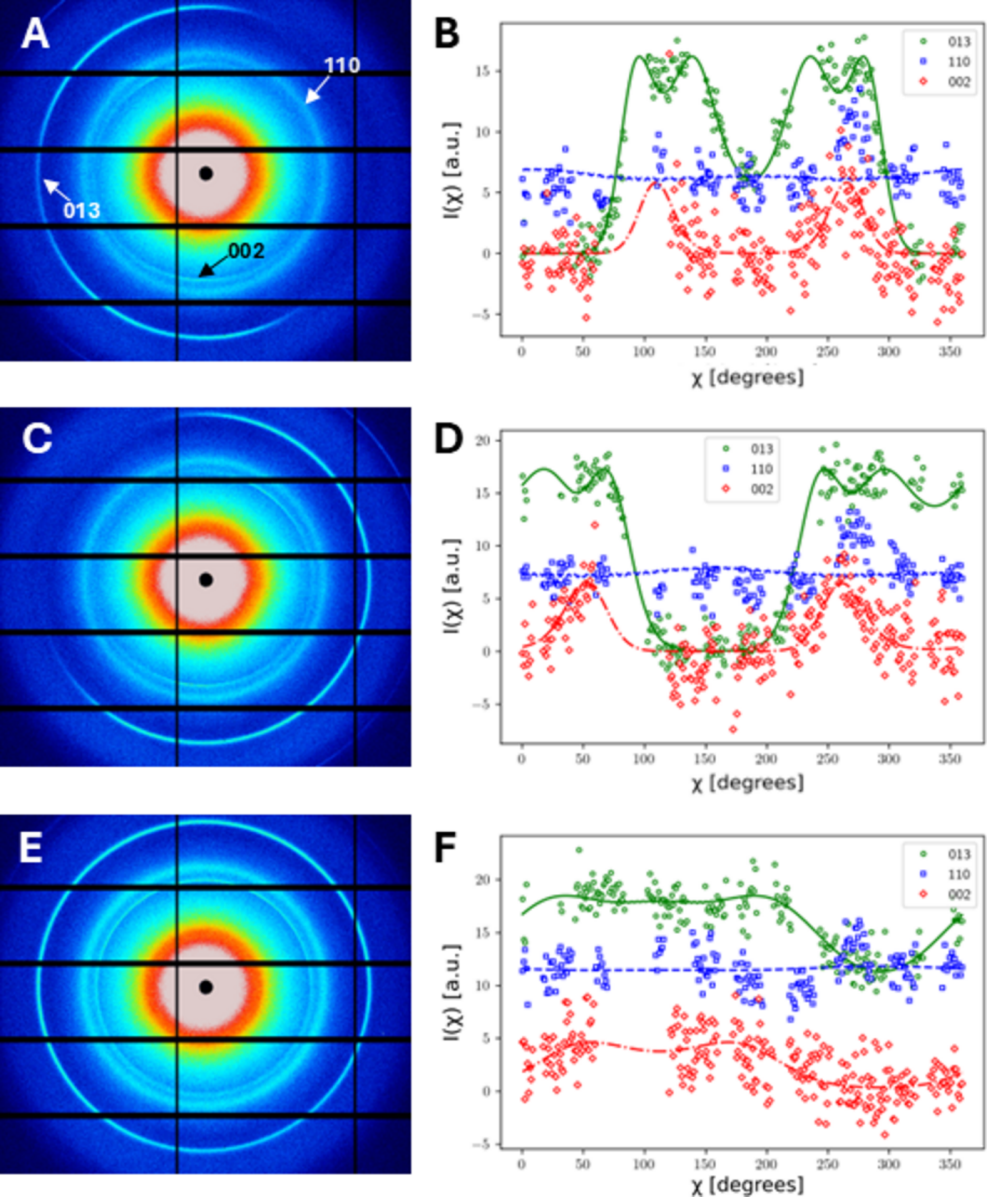
Experimental data and model fits from cuticle samples in the L1 geometry. (Left) Experimentally recorded X-ray patterns from three samples. The (002), (110) and (013) reflections from chitin fibres are indicated by arrows in panel (*a*). (Right) Azimuthal intensity profiles *I*(χ) of the three reflections. Panels (*a*) and (*b*) show data for sample S01, panels (*c*) and (*d*) for sample S02 and panels (*e*) and (*f*) for sample S03. The colour coding is as follows: axial reflection 002, red; equatorial reflection 110, blue; 013, example of the general case, green. Open circles, squares and diamonds are experimental data and solid lines are the model fits.

While a perfect L1 orientation in cuticle samples, along with a Bouligand stack, leads to uniform intensity across the full azimuthal range (χ = 0–360°), small deviations (tilt angles α and/or β ≃ 10–20°) from the condition where all fibres are in the *x*–*y* plane [(α, β) = (0°, 0°)] cause significant angular asymmetry in the *I*(χ) profiles. This is illustrated in the fitted profiles shown in Fig. 4 ▸; cuticle samples S01 [Figs. 4 ▸(*a*) and 4 ▸(*b*)] and S02 [Figs. 4 ▸(*c*) and 4 ▸(*d*)] show significant angular anisotropy in the experimental 2D X-ray patterns and 1D azimuthal intensity profiles, resulting from tilt angles (α, β) ≃ (−2.0°, 24.0°) and (−6.8°, −18.1°), respectively. In contrast, the nearly flat profile in sample S03 [Figs. 4 ▸(*e*) and 4 ▸(*f*)] corresponds to both (α, β) tilt magnitudes being small (<10°) at (7.6°, 3.9°), as summarized in Table 2 ▸.

### 4.2. Nanobeam for sublamellar Bouligand structure

**Figure 5:**
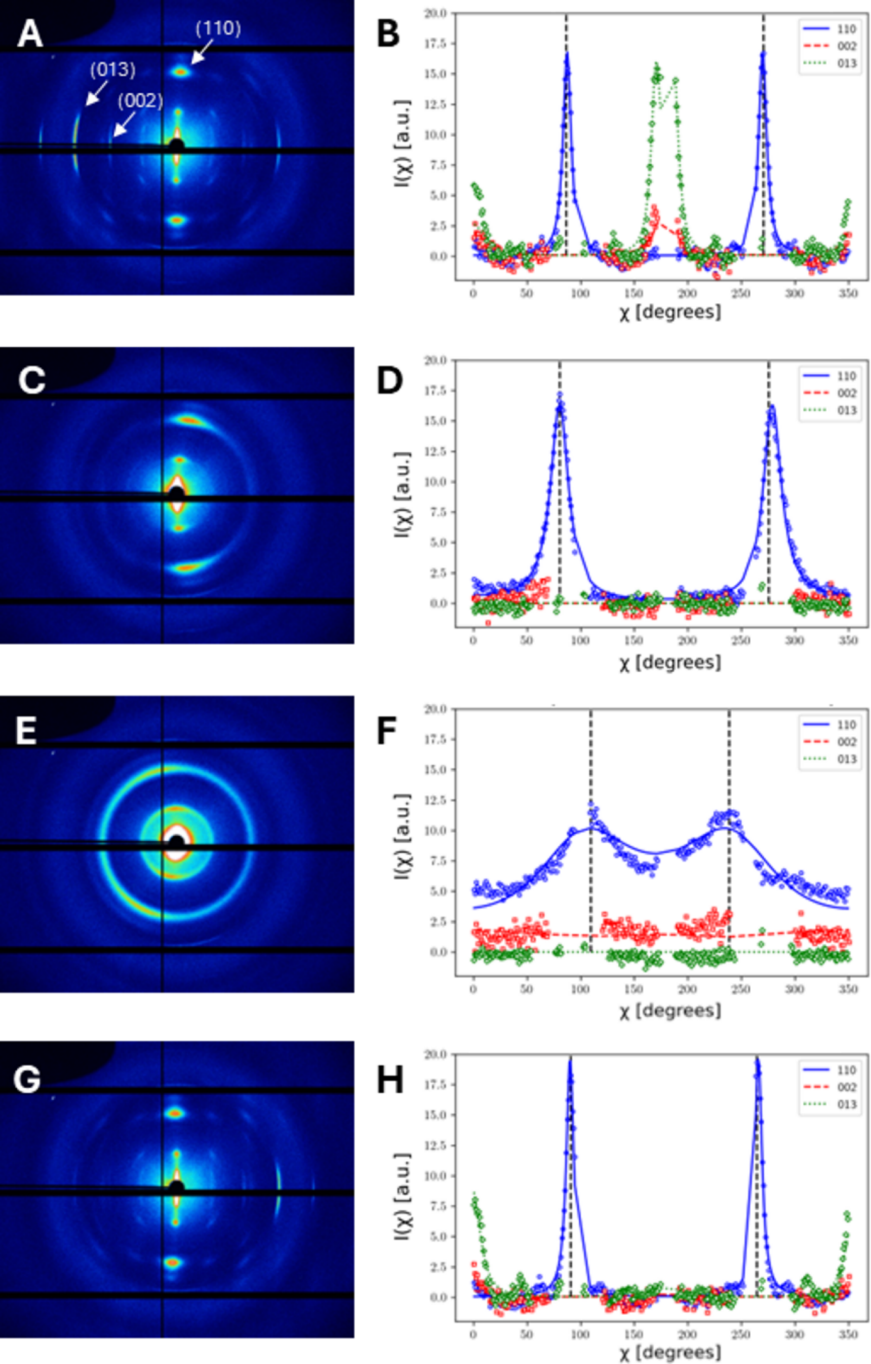
Experimental data and model fits from a single cuticle sample in the L3 geometry. These data were taken from a line scan along the cuticle thickness in the endocuticle region. (*a*), (*c*), (*e*) and (*g*) Experimentally recorded X-ray patterns from different layers in a single lamella. (*b*), (*d*), (*f*) and (*h*) Azimuthal intensity profiles *I*(χ) integrated from the (002), (110) and (013) reflections. The arrows in panel (*a*) indicate the three chitin reflections (002), (110) and (013). In the 1D profiles, open circles, squares and diamonds are experimental data and solid lines are the model fits, with red denoting the (002) reflection, blue (110) and green (013).

By using a smaller nano-focus beam (P06, DESY), the internal fibre structure and orientation of the sublamellae in the Bouligand arrangement can be probed. We scanned the middle region of the cross-sectional area of a cuticle sample in L3 geometry and collected X-ray patterns through the whole thickness of ∼300 µm with a step size of 3 µm (along the cuticle thickness direction). Fig. 5 ▸ shows examples of the experimental X-ray patterns (left) and azimuthally integrated intensity profiles *I*(χ) (right) for the (110), (002) and (013) reflections (scattered data in blue, red and green, respectively). These patterns are taken from a line scan along the cuticle thickness in the endocuticle region, ∼60 µm, which corresponds to spanning a lamella in the endocuticle of this sample. The (*hk*0) class of reflections, *i.e.* (110), remains visible throughout the scan, but changes are observed in the peak position and peak width, and the separation of the two peaks deviates from 180° quite significantly, which is also observed in previous work (Stribeck, 2009 ▸; Paris & Müller, 2003 ▸; Zhang *et al.*, 2016 ▸). This is due to the broadening of the intersection of (110) rings with the Ewald sphere as fibres rotate around the vertical axis (during the rotation β) in the Bouligand pattern. However, the (0*kl*) reflections including (002) and (013) are not always visible [Figs. 5 ▸(*c*)–5 ▸(*f*)]. This is because the (002) and (013) reflections no longer intersect the Ewald sphere after about a 10–20° tilt in β and cannot contribute to the intensity. Therefore, fitting the (110) reflection simultaneously with the (0*kl*) reflections is needed. It would in principle be possible to add more reflections [*e.g.* (004) or (020)] to the fit, but here we focus on the three most prominent and complementary ones.

**Figure 6:**
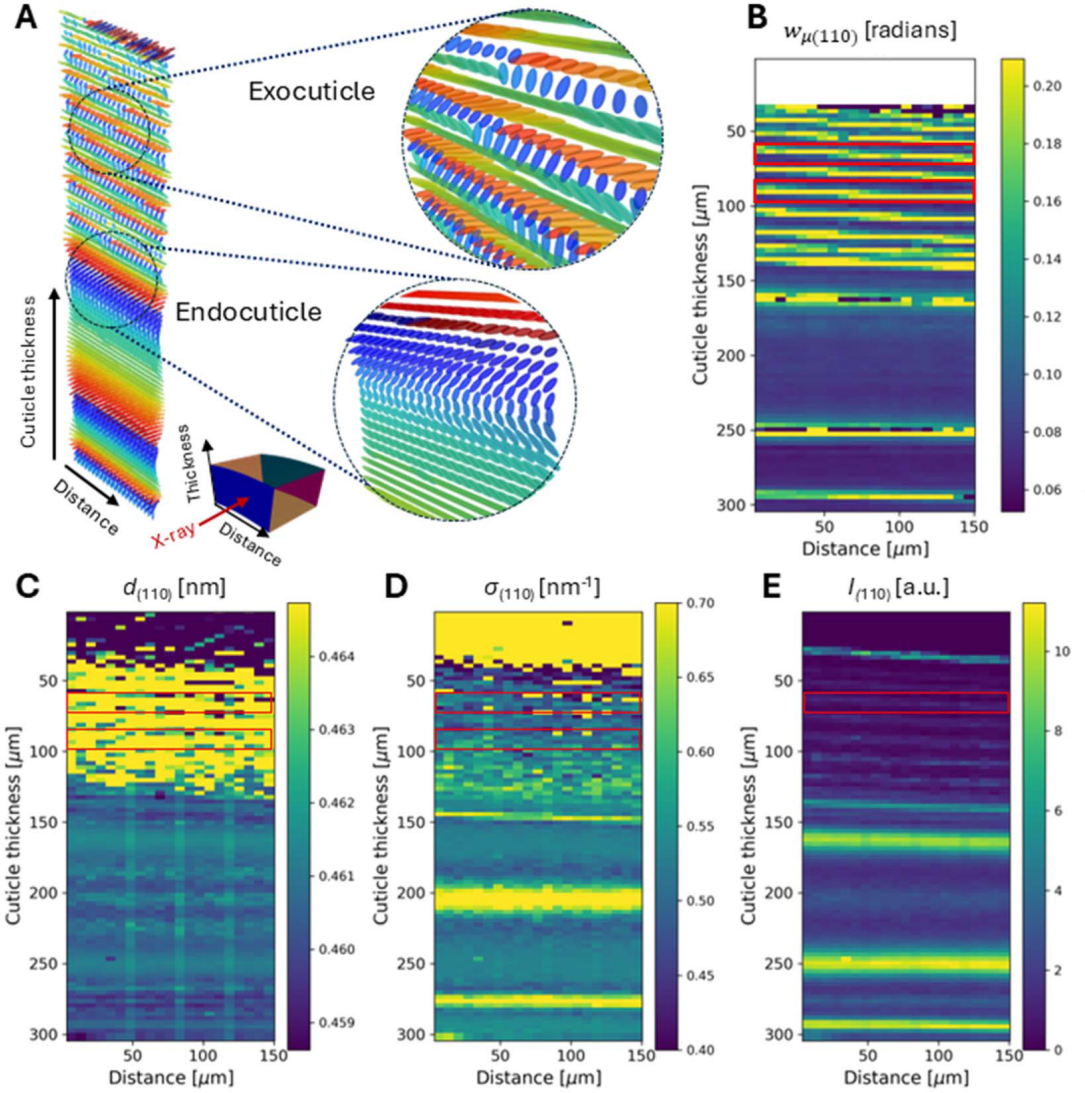
Reconstruction of the sublamellar Bouligand structure in cuticle from experimental data. (*a*) Fibre orientation in the Bouligand structure, calculated using the diffraction model. The fibre orientation is determined by the tilt angles (α, β), and the oblate ellipsoids’ degree of elongation is inversely proportional to fibril misalignment. The enlargements show the chitin fibrils in the exocuticle and endocuticle. The schematic diagram at the bottom shows the direction and geometry of the sample. (*b*) Angular width distribution *w*_μ(110)_ for the (110) reflection calculated from the azimuthal intensity. (*c*) Distribution of the lattice spacing *d*_(110)_. (*d*) Width of the radial peak σ_(110)_. (*e*) Radial intensity *I*_(110)_ of the (110) reflection. Red rectangles outline sections in the exocuticle. The X-ray scan was performed in the middle of the cross section of the sample in L3 geometry, covering the full cuticle thickness. Scan range ∼150 µm along the width and ∼300 µm along the thickness. Step size 7 µm in the width direction and 3 µm in the thickness direction.

To determine the fibril orientation, we assumed each sublamella has a single fibre direction and modelled the diffraction signals using the single fibril diffraction intensity formula [equation (4)] with *w*(γ) as a Dirac δ-function. As in the above section for L1 geometry modelling, the tilt angles (α, β) were shared across the three reflections. The matching of the fits (lines in Fig. 5 ▸) with the experimental data (scattered data points in Fig. 5 ▸) provides evidence that our model can be used to reconstruct orientations and tilts of fibrils at the sublamellar level. We then applied the model to the full X-ray scan of this cuticle sample and quantified the orientation parameters of each sublamella to reveal the fibril arrangement throughout the cuticle thickness. As the (002) and (013) reflections are not visible at all points in the scan due to large tilt angles and the module gaps on the detector, only the results for the (110) reflection are shown in Fig. 6 ▸.

For the graphical representation in Fig. 6 ▸(*a*) using *MayaVi* (Ramachandran & Varoquaux, 2011 ▸), each voxel in the 2D scan was rendered as an oblate ellipsoid whose degree of elongation was inversely proportional to the fibril misalignment *w*_μ(110)_ [Fig. 6 ▸(*b*)] in the voxel (shorter, more spherical ellipsoids correspond to less-aligned fibrils) and the 3D direction determined by (α, β). The results show a clear lamellar structure at a slight but nonzero angle to the horizontal, with thin exocuticle (∼15 µm) and thick endocuticle (∼50–90 µm) lamellae. Interestingly, the interfaces between lamellae show a greater fibril misalignment; this can arise from a combination of (i) a rapidly varying fibre orientation change convolved with through-thickness sampling and sample-tilt artefacts leading to higher apparent misalignment, and (ii) genuine misalignment at the sublamellar level, as far as these can be conceptually distinguished without diffraction or tomographic measurements (a larger spatial voxel of 50–200 µm in size would lead to apparently misaligned fibrils within the central part of each lamella, due to averaging of fibrils from different sublayers).

To study further the fibril structural change through the cuticle thickness and the ultrastructural differences in exo- and endocuticle, we integrated the X-ray patterns in the radial direction. From the radially integrated *I*(*q*) profiles, the lattice spacing of the (110) reflection *d*_(110)_ = 2π/*q*_(110)_ [*q*_(110)_ is the radial peak centre of the (110) reflection] was calculated [Fig. 6 ▸(*c*)]. Due to the overlapping of the broad (110) reflection with multiple other reflections, the radial characteristics of the (110) reflection [radial peak position *q*_0(110)_ and *dq*_0(110)_] were obtained by fitting the (110) reflection along with significant neighbouring reflections in a ±1.5 nm^−1^ range [*e.g.* (002), (012), (100), (101), (120) and (111)]; all radial peaks were allowed a small level of variability (±2%) in location, taking into account the typical pre-strains observed in the tissue. While the intensities of these reflections are low, excluding them will artificially induce shifts and apparent pre-strain changes in the (110) radial peak position and thus need correction. The results, shown in Figs. 6 ▸(*c*)–6 ▸(*e*) and correlated with the angular fit parameters [Figs. 6 ▸(*a*) and 6 ▸(*b*)], show an intricate spatial variation, not only in the expected angular change (α and β) but also in the angular dispersion [*w*_μ(110)_], lattice spacing [*d*_(110)_] and radial peak width [σ_(110)_], suggesting lattice distortions and strain heterogeneity throughout the cuticle. In the exocuticle, larger strain heterogeneity [through the radial peak width σ_(110)_] correlates with regions with lower lattice spacing *d*_(110)_ [red rectangles, Figs. 6 ▸(*c*) and 6 ▸(*d*)]. Further, by comparing *w*_μ(110)_ and σ_(110)_ [red rectangles, Figs. 6 ▸(*b*) and 6 ▸(*d*)], a positive correlation is observed. The physical interpretation is that the increased strain heterogeneity arises from the varying lattice spacings of the chitin microfibrils at different angles to the main fibre axis, which broaden the *I*(*q*) peak. Spatial variation is also observed in the thicker endocuticle, but the correlations between *d*_(110)_ and σ_(110)_ are not as consistent.

## 5. Discussion

This work presents and validates a general diffraction model for anisotropic materials with fibre symmetry in the scattering volume, extending beyond the special cases described in our earlier studies and demonstrating how to analyse depth-averaged 3D orientation and other crystallographic information. Previously, we focused on configurations aligned at 0° and 90° to the fibre axis, corresponding to the (002) and (110) reflections, respectively. Here, we present the full solution for arbitrary sample orientations, including the intermediate (0*kl*) reflections, such as the (013) reflection. (0*kl*) encompasses all possible reflections between the axial (0°) and equatorial (90°) limits, enabling its use in concurrent fitting of multiple reflections in fibre materials where the orientation varies spatially or during scanning. We also present a unified way to extract initial parameter estimates like orientation (α, β) and intrinsic angular dispersion *w*_μ_ from the azimuthal intensity characteristics. Together, our approach enables a framework for interpreting WAXD scans of anisotropic heterogeneous materials with nanoscale fibre symmetry, *e.g.* complex bio­logical and synthetic fibrous systems, and it could also be embedded in more complex methods for reconstruction or data-based/AI approaches (Sun *et al.*, 2023 ▸).

Pioneering work on cellulose fibrils (Lichtenegger *et al.*, 1999 ▸; Lichtenegger *et al.*, 2003 ▸; Paris & Müller, 2003 ▸; Ogurreck *et al.*, 2010 ▸, 2013 ▸) used the equatorial (110) reflection, under fibre symmetry assumptions, to determine the 3D orientation (α, β in our terminology) from the profile characteristics. However, the degree of intrinsic angular dispersion (*w*_μ_ in our model), the unit-cell dimensions, strain heterogeneity (*q*_0_, *dq*_0_) or other reflections were not explicitly considered. In our earlier work on chitin fibrils (Zhang *et al.*, 2016 ▸), we developed analytical models for (110) fibre-symmetric diffraction and, later, axial (002) stress-induced axial fibril strains (Zhang *et al.*, 2017 ▸), but only in near-planar cases.

With higher spatial resolution of X-ray beams at third- and fourth-generation synchrotron sources, orientational averaging approximations valid for large scattering volumes no longer hold for scanning textured anisotropic materials in many cases. Many biological anisotropic nanomaterials like cuticle and bone occupy an intermediate position in terms of texture, between polycrystalline inorganic materials with discrete Bragg spots and isotropic (albeit fibrous) hydrogels. The basic scattering units in such cases are nanometre-sized fibrils (3–4 nm diameter for chitin fibrils and a similar range for collagen microfibrils), but lateral crystalline co-registry over tens to hundreds of nanometres is not usual, so even within *e.g.* a volume of ∼1 µm^3^ one would expect a preferred orientation for chitin fibre but fibre symmetry around the fibril *c* axis. The lack of discrete separable Bragg spots due to the small size of the scattering domains limits the application of other diffraction tomographic methods but has the contrary advantage that the broadened diffraction signal from many fibres can be detected even when they do not perfectly satisfy the Ewald diffraction condition; this is parameterized in our model by the angular width *w*_μ_.

Recent work by Carlsen *et al.* (2025 ▸) and Frewein *et al.* (2024 ▸) tackled this question in the field of texture tomography in complementary ways – the former by considering broadening of the perfect crystallite diffraction spots to Gaussian basis functions on the sphere, and the latter by incorporating orientation distribution functions at each voxel, which build patterns out of perfect crystallites, and using generalized spherical harmonics. While the present paper does not include or attempt tomographic reconstruction, we note that we consider the fibril as the basic scattering unit (*i.e.* one level of coarse graining above individual crystallites), so our approach is likely to apply to more intrinsically disordered (or at least axially rotated) systems than the above and [for the same reasons as given by Frewein *et al.* (2024 ▸)] incorporate multiple reflection fitting. An alternative recent approach to the question of determining nanofibre or particle orientation without sample rotation is energy-dispersive Laue diffraction (EDLD), which can obtain position-resolved texture information without rotation using a white beam and a pixellated area detector (Sakr *et al.*, 2024 ▸). The closest prior similar work we are aware of is that by Ogurreck *et al.* (2010 ▸, 2013 ▸), who analysed the azimuthal diffraction from the cellulose microfibrils in softwood cells (tracheids), building on prior work by Lichtenegger *et al.* (2003 ▸) and Paris & Müller (2003 ▸) among others. In contrast to their analyses, we do not consider the higher-level tubular tracheid structure, but the planar Bouligand structure found in cuticle. Further, we report values for both the azimuthal peak locations and the widths of the (00*l*), (*hk*0) and (*hkl*) reflections (Table 1 ▸), show how tilt parameters may be estimated from peak shifts, relative intensity changes and half-widths of the peaks, and describe the components of the general fitting function. These have not been reported in prior work, to the best of our knowledge.

The model is tested and validated by reproducing the expected Bouligand spiralling pattern for the principal fibre direction (Fig. 6 ▸), which also reveals subtler features like increased variability in angular direction at the edges of lamellae and gradients in lattice spacing. These correlations are different between the outer exo- and inner endocuticle. In the stiffer highly mineralized exocuticle, the radial peak width σ_(110)_, linked to micro-strain broadening, is lower for larger lateral lattice spacings [red rectangles, Figs. 6 ▸(*c*) and 6 ▸(*d*)]. The fibrils with smaller lattice spacing *d*_(110)_ are laterally (side-by-side) compressed; their increased σ_(110)_ is linked to an associated compressive microstrain. Our previous work (Zhang *et al.*, 2020 ▸) showed that the axial lattice spacing *d*_(002)_ was smaller in the exo- than the endocuticle. The higher average *d*_(110)_ in the exocuticle [Fig. 6 ▸(*c*)] is consistent with these prior results, where axially compressed (but laterally expanded) fibrils in the exocuticle coexist with longer thinner fibrils in the endocuticle. In contrast to our previous work (Zhang *et al.*, 2020 ▸), here the 3D model enables smooth extraction across the entire tissue with no gaps in coverage. Due to the limited time available at synchrotron sources, these results were obtained on only a single sample set and would need to be biologically validated with statistically significant sample sizes.

**Figure 7:**
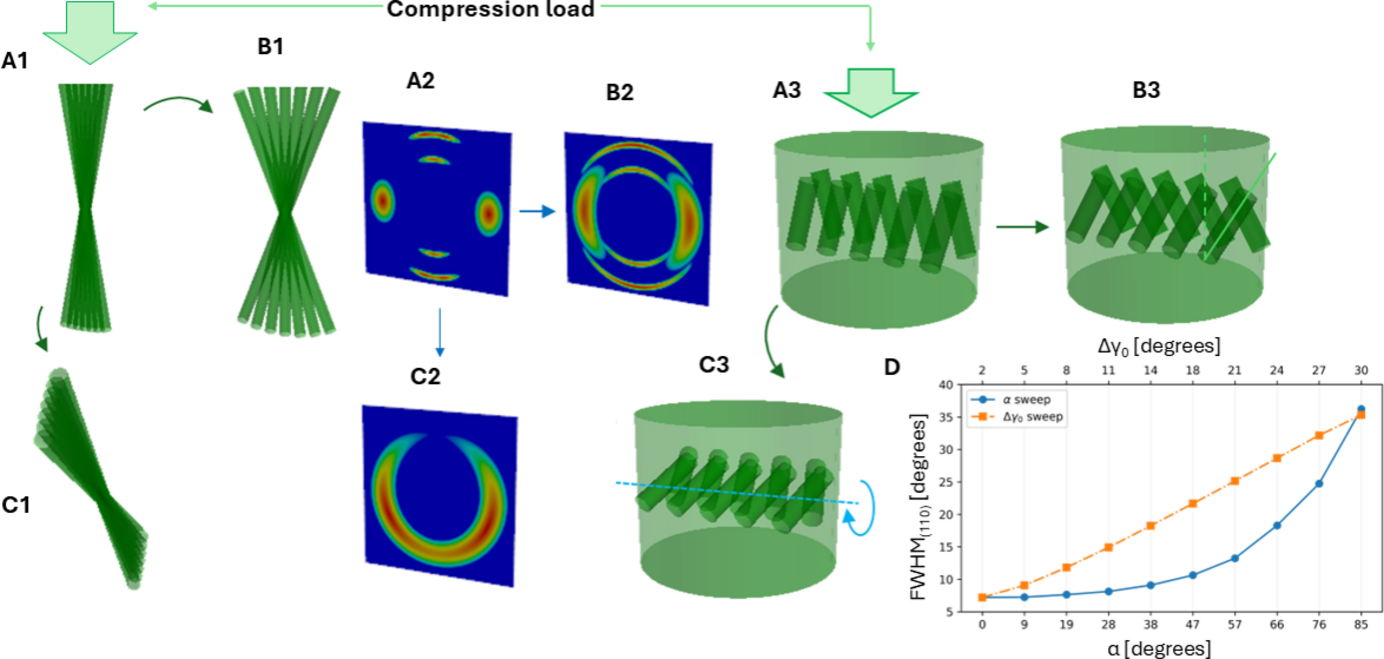
Distinguishing modes of time-resolved fibrillar mechanics and the response of chitin fibres to compressive load (vertical arrows). Chitin fibres oriented initially along the loading axis [(*a*1) original fibril array, (*a*3) original fibres in the tissue] may broaden the in-plane distribution [(*a*1)→(*b*1), (*a*3)→(*b*3)] or tilt as a block out of the plane [(*a*1)→(*c*1), (*a*3)→(*c*3)]. The X-ray diffraction patterns (and azimuthal plots) would show a similar broadening effect in both cases [(*a*2)→(*b*2), (*a*2)→(*c*2)] although at different rates. (*d*) FWHMs of the (110) reflection at the two conditions at a given *w*_μ_. The orange plot shows the increase in the in-plane fibril distribution (Δγ_0_) and the blue plot shows the increase in the fibril tilt (α).

The ability to extract 3D fibril orientations without sample rotation is essential when studying the time-resolved or kinetic behaviour of nanofibrous materials, where the X-ray beam needs to be localized to a specific region during a kinetic process. For example, applying a load to a lamellar fibril array with a range of orientations can lead to a combination of rotation, extension or narrowing of the angular width of the distribution. Due to the 3D structure, analysis of the 2D angular distribution by itself will not be sufficient, as shown by the simulated example in Fig. 7 ▸. Considering the angular width of the (110) reflection, an apparent broadening is observed under a compressive load when a single fibre rotates away from the detector plane. Using standard 2D orientation measures, this behaviour would be interpreted as a compression-induced broadening of fibre distribution around the loading direction, while the true 3D behaviour is that of a single fibre rotating away from the loading axis.

A benefit of the general model is that combined fitting of multiple reflections increases the reliability of the fit and the extraction of orientation and related crystallographic parameters in situations where the Ewald condition is not being met for some of the reflections. For example, when tracking a single fibre orientation across the lamellae in Fig. 5 ▸, the (002) and (013) reflections are visible and rapidly varying around β = π/2, where the azimuthal (110) profile changes are minimal, while near β = 0 and π, (002) and (013) are absent and the (110) azimuthal profile shows significant variability, going from relatively sharply peaked to quite broad as β → 0 or π. Thus, using all three reflections simultaneously makes the fit sensitive to the tilt parameters across all possible conditions.

The code for fitting and graphical rendering of the reflections is being developed in a graphical user interface-based package, following existing templates like *D+* (Balken *et al.*, 2023 ▸), and will be released in upcoming work.

In summary, we have described quantitatively a framework for the diffraction analysis of fibre-symmetric nanofibres inside composite materials, considering both individual fibres and planar arrays, and multiple crystallographic reflections – axial, equatorial and the intermediate class. The model has been validated on a prototypical chitin cuticle composite. The generality of the formulation allows it to be extended to other materials with fibre symmetry, including cellulose, bone-mineralized fibrils and other natural composite materials, and makes it amenable to possible embedding into tomographic and data-based methods.

## Supplementary Material

Detailed calculations, plus additional figure and table. DOI: 10.1107/S2052252525009686/ro5047sup1.pdf

## Figures and Tables

**Table 1:** Expressions for the azimuthal peak positions, the angles at HWHM from the azimuthal profiles below 180° and the intensity ratios of the two peaks in the azimuthal profiles for the (00*l*), (*hk*0) and (*hkl*) reflections *I*_u_ is the intensity at the azimuthal angle χ ≃ 90° for the (00*l*) and (*hkl*) reflections and χ ≃ 180° for the (*hk*0) reflection. *I*_l_ is the intensity at the azimuthal angle χ ≃ 270° for the (00*l*) and (*hkl*) reflections and χ ≃ 360°/0° for the (*hk*0) reflection. Derivations are given in the supporting information, Sections S5.1 for (00*l*), S5.2 for (*hk*0) and S5.3 for (*hkl*).

Reflection	Peak position (radians)	Angle at HWHM (radians)	Intensity ratio *I*_u_/*I*_l_ (no units)
(00*l*), μ ≡ μ_(00*l*)_ = 0, *w*_μ_ ≡ 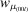	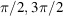	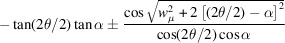	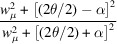
			
(*hk*0), μ ≡ μ_(*hk*0)_ = π/2, *w*_μ_ ≡ 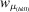	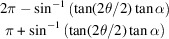	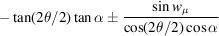	1
			
(*hkl*), μ ≡ μ_(*hkl*)_, *w*_μ_ ≡ 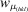	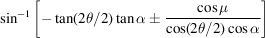	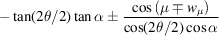	Variable, see discussion after equation (S26) in the supporting information

**Table 2:** Fit parameters for L1-oriented cuticle samples in Fig. 4 Values reported are mean ± standard deviation.

Sample	α (°)	β (°)
S01	−1.96 ± 0.54	23.95 ± 1.17
S02	−6.80 ± 0.17	−18.06 ± 0.27
S03	7.58 ± 0.76	3.88 ± 0.66
