# Supplementary material for: Anisotropic diffraction of materials with fibre symmetry: application to chitin cuticle
Source: IUCrJ. 2026 Jan 1;13(Pt 1):63–76. doi: 10.1107/S2052252525009686 (PMC12809525; doi:10.1107/S2052252525009686)
Supplement: Supplementary file 1 [file m-13-00063-sup1.pdf]

IUCrJ

Volume 13 (2026)

Supporting information for article:

Anisotropic diffraction of materials with fibre symmetry: application to chitin cuticle

Yanhong Wang, Tim Snow, Nick Terrill and Himadri Shikhar Gupta

S1. Fit process:

The fit procedure minimizes the residual between I(χ) and the model prediction. I(χ) is experimentally calculated by averaging over a radial range around the reciprocal lattice wavevector value q_0, for fixed χ. To calculate the model prediction at a given (q, χ) value for scattering vector magnitude and azimuthal angle χ, and for a fibre at angles (α, β), the function calculates the corresponding reciprocal space vectors in the fibre frame (Q_x^F, Q_y^F, Q_z^F) using the relations in Equation 5 main text. For single fibre diffraction (L3-geometry), these fibre-frame reciprocal lattice vectors are used in the equation for $\Theta(\mathbf{Q^F})$ and $\delta_\mu(\mathbf{Q^F})$. By radially averaging the result over the same q-range around each reciprocal lattice wavevector value q_0, I(χ) is obtained. For the isotropic planar orientation distribution in L1, a discrete integral averages the signal from individual fibres at γ distributed uniformly from 0° to 180° to give the model intensity. The minimization uses the *lmfit minimize*() function, with the residual of the model prediction and experimental data combined across (002), (013) and (110) profiles. In some cases, one or more of the reflections have no significant nonzero intensity (or model prediction) in the unmasked regions (e.g. L3, **Fig 5D**: (002) and (013)). Here, though the total residual is calculated and used, the parameter change leading to residual minimization is in practice driven by changes in the (110) model prediction in such cases.

The explicit formula for the intensity for each case will involve multiple terms of the type shown in Eqn. (5) incorporated into the Θ- and related functions and will be lengthy to write down as a one-line expression. However, the sequence of function calls can be described:

1. For a given (q, χ) calculate the equivalent reciprocal-space vector components (Q_x, Q_y, Q_z) on the Polanyi circle **(Figure S1 C**, **Table S1** last row)
2. Transform the reciprocal space coordinates to fibre-frame coordinates using the relations in (S3) and calculate Θ.
3. For the (*hkl*) reflection (angle μ) for a single fibril oriented vertically ($\gamma = 0$) apply Eq. (1) in main text to calculate δ_μ from Θ; in case of a planar array of fibrils oriented at different angles γ, apply Eq. (4) to integrate δ_μ across different γ.
4. Step 2-3 can be combined by using (S1)-(S2) which contains the geometrical transformation.

S2. Intensity expressions:

$$I_\mu(\boldsymbol{q}) = \int_{-\frac{\pi}{2}}^{\frac{\pi}{2}} w(\gamma;\{\gamma_0,\Delta\gamma_0\})\delta_\mu\big(\boldsymbol{Q^F}(q,\gamma;\alpha,\beta,2\theta);\mu,\gamma\big) \quad \text{(S1)}$$

with Q^F given by Eq. (S3) and Table S1, last line

$\Theta(q,\gamma;\alpha,\beta,2\theta)$, used as the argument for the angular peak function P_θ in δ_μ, is

$$\Theta(q,\gamma;\alpha,\beta,2\theta) = \tan^{-1}\left(\frac{\boldsymbol{q^L}.\boldsymbol{\Lambda}_2}{\sqrt{(\boldsymbol{q^L}.\boldsymbol{\Lambda}_1)^2+(\boldsymbol{q^L}.\boldsymbol{\Lambda}_3)^2}}\right) \quad \text{(S2)}$$

where vectors $\boldsymbol{\Lambda}_1, \boldsymbol{\Lambda}_2, \boldsymbol{\Lambda}_3, \boldsymbol{q^L}$ are defined in Table S1:

Table S1

Vector	Expression
$\mathbf{\Lambda}_1$	$(cos\,\beta\,,0,-sin\,\beta)$
$\mathbf{\Lambda}_2$	$(\sin\alpha\sin\beta\,,\cos\alpha\,,\sin\alpha\cos\beta)$
$\mathbf{\Lambda}_3$	$(\cos\alpha\sin\beta\,,-\sin\alpha\,,\cos\alpha\cos\beta)$
$\boldsymbol{q}$	$q\left(\cos\frac{2\theta}{2}\cos\chi\,,\cos\frac{2\theta}{2}\sin\chi\,,\sin\frac{2\theta}{2}\right)$

α,β are the tilt angles of fibre out of the x-y plane (**Figure 2D**), χ is the detector azimuthal angle, q is the scattering vector.

S3. Transformation between lab and fibre coordinates:

The reciprocal space coordinates in the fibre system $Q_{x,y,z}$ in terms of lab reciprocal space coordinates $Q_{x,y,z}{}^{L}$ are

$$\begin{bmatrix} Q_x^F \\ Q_y^F \\ Q_z^F \end{bmatrix} = \begin{bmatrix} \cos\beta & 0 & -\sin\beta \\ \sin\alpha\sin\beta & \cos\alpha & \sin\alpha\cos\beta \\ \cos\alpha\sin\beta & -\sin\alpha & \cos\alpha\cos\beta \end{bmatrix} \begin{bmatrix} Q_x \\ Q_y \\ Q_z \end{bmatrix} \qquad \text{(S3)}$$

For the coordinates on the Polanyi circle, Q_x etc. are replaced by $\boldsymbol{q}$ in Table S1.

S4. Graphical rendering of Θ, (0kl) intensity and Ewald construction

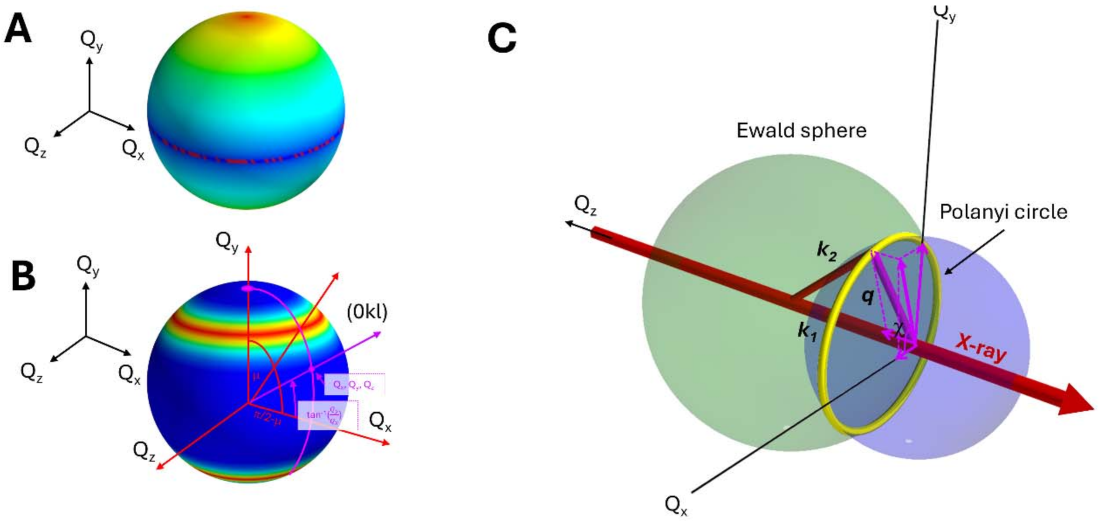

Figure S1: *3D rendering of A:* $\Theta(\boldsymbol{Q})$ *and B:* $\delta_\mu(\boldsymbol{q};\mu,0)$ *using* $\Theta(\boldsymbol{Q})$ *in the peak function, showing the upper and lower fibre-symmetric rings.* $\mu=\frac{\pi}{4}$ *to show the ring more clearly than a narrower (013) angle of μ~10.3°. The magenta arcs show the inverse tangent meaning of* Θ *as an angle, which needs to match* $\frac{\pi}{2}-\mu$ *to maximise the value of the peak function* P_θ *when the reciprocal space vector is on the high intensity ring corresponding to the (0kl) peak. C: Ewald sphere construction, showing incident beam (red), incident and scattered wavevectors* $\boldsymbol{k_1}$ *and* $\boldsymbol{k_2}$*, and scattering vector* $\boldsymbol{q}$*. The Ewald sphere is*

shown in light green; the intersection with a sphere of fixed radius q (Polanyi circle) is shown in yellow; pink arrows are the reciprocal space components of $\boldsymbol{q}$.

S5. Peak positions and FWHM/HWHMs of reflections:

To estimate peak orientations and angular dispersions from the peak positions and FWHMs of the different reflections, an analysis in the same spirit as earlier work by Stribeck [1] (building on Polanyi's prior work[2]) is carried out. The angular term P_θ in the intensity expression is $P_\theta = \frac{1}{1+\left(\frac{\Theta(\boldsymbol{Q}^{\boldsymbol{F}})-\left(\frac{\pi}{2}-\mu\right)}{w_\mu}\right)^2}$, which is maximum when the term in parenthesis is minimum. We consider the case where $\alpha \neq 0, \beta = 0$ as discussed in the main text. Expressing $\Theta(\boldsymbol{Q}^{\boldsymbol{F}})$ in terms of $q, \chi, \frac{2\theta}{2}$ on the Polanyi circle (Table S1), and the tilt angles, we have

$$\Theta(q, \chi; \alpha, 0, 2\theta) = \tan^{-1}\left(\frac{\sin\frac{2\theta}{2}\sin\alpha+\cos\frac{2\theta}{2}\sin\chi\cos\alpha}{\sqrt{\left(\cos\frac{2\theta}{2}\cos\chi\right)^2+\left(\sin\frac{2\theta}{2}\cos\alpha-\cos\frac{2\theta}{2}\sin\chi\sin\alpha\right)^2}}\right) \quad \text{(S4)}$$

S5.1. (00l)

For (00l), $\mu = 0$ and using $\tan^{-1}\frac{1}{x} = \frac{\pi}{2} - \tan^{-1}x$ we have

$$\Theta_{(00l)}\left(q_{(00l)}, \chi; \alpha, 0, 2\theta\right) = \tan^{-1}\left(\frac{\sqrt{\left(\cos\frac{2\theta}{2}\cos\chi\right)^2+\left(\sin\frac{2\theta}{2}\cos\alpha-\cos\frac{2\theta}{2}\sin\chi\sin\alpha\right)^2}}{\sin\frac{2\theta}{2}\sin\alpha+\cos\frac{2\theta}{2}\sin\chi\cos\alpha}\right) \quad \text{(S5)}$$

For the extrema χ values of this function it is sufficient to find the extrema of the argument. The derivative of the term within parenthesis, with χ, is

$$-2\cos^2\frac{2\theta}{2}.\frac{\cos\chi\left(\sin\chi\cos^2\alpha+\tan\frac{2\theta}{2}\cos\alpha\sin\alpha\right)}{\left(\sin\frac{2\theta}{2}\sin\alpha+\cos\frac{2\theta}{2}\sin\chi\cos\alpha\right)\sqrt{\left(\cos\frac{2\theta}{2}\cos\chi\right)^2+\left(\sin\frac{2\theta}{2}\cos\alpha-\cos\frac{2\theta}{2}\sin\chi\sin\alpha\right)^2}} \quad \text{(S6)}$$

which, for α in the range of tilts up to but not equal to $\frac{\pi}{2}$, is zero for $\chi = \frac{\pi}{2}, \frac{3\pi}{2}$ as expected.

Evaluating the $\Theta(\boldsymbol{Q}^{\boldsymbol{F}})$ term at $\chi = \frac{\pi}{2}, \frac{3\pi}{2}$ gives

$$\Theta_{(00l)}\left(q_{(00l)}, \frac{\pi}{2}; \alpha, 0, 2\theta\right) = \tan^{-1}\left(\frac{\sin\left(\frac{2\theta}{2}-\alpha\right)}{\cos\left(\frac{2\theta}{2}-\alpha\right)}\right) = \frac{2\theta}{2} - \alpha \quad \text{(S7.1)}$$

$$\Theta_{(00l)}\left(q_{(00l)}, \frac{3\pi}{2}; \alpha, 0, 2\theta\right) = \tan^{-1}\left(\frac{\sin\left(\frac{2\theta}{2}+\alpha\right)}{\cos\left(\frac{2\theta}{2}+\alpha\right)}\right) = \frac{2\theta}{2} + \alpha \quad \text{(S7.2)}$$

The peak intensity ratios are equal to the ratios of P_θ

$$\frac{P_\theta\left(\frac{\pi}{2}\right)}{P_\theta\left(\frac{3\pi}{2}\right)} = \frac{1+\left(\frac{2\theta}{2}+\alpha\right)^2}{1+\left(\frac{2\theta}{2}-\alpha\right)^2} \quad \text{(S8)}$$

The full width at half maxima can be obtained by solving $P_\theta(\chi) = \frac{1}{2}P_\theta\left(\frac{\pi}{2}\right)$ for χ, or

$$\left(\frac{1}{w_\mu}\tan^{-1}\left(\frac{\sqrt{\left(\cos\frac{2\theta}{2}\cos\chi\right)^2+\left(\sin\frac{2\theta}{2}\cos\alpha-\cos\frac{2\theta}{2}\sin\chi\sin\alpha\right)^2}}{\sin\frac{2\theta}{2}\sin\alpha+\cos\frac{2\theta}{2}\sin\chi\cos\alpha}\right)\right)^2 = 1+2\left(\frac{\frac{2\theta}{2}-\alpha}{w_\mu}\right)^2 \quad \text{(S9)}$$

which simplifies to

$$\cos^2\frac{2\theta}{2}+\left(\sin\frac{2\theta}{2}\cos\alpha-\cos\frac{2\theta}{2}\sin\chi\sin\alpha\right)^2 = \tan^2\Delta\left(\sin\frac{2\theta}{2}\sin\alpha+\cos\frac{2\theta}{2}\sin\chi\cos\alpha\right)^2$$

(S10)

where $\tan\Delta = \tan\sqrt{w_\mu^2+2\left(\frac{2\theta}{2}-\alpha\right)^2}$.

The above has the solutions

$$\sin\chi_\pm^{(00l)} = \frac{-\sin\frac{2\theta}{2}\sin\alpha\pm\cos\sqrt{w_\mu^2+2\left(\frac{2\theta}{2}-\alpha\right)^2}}{\cos\frac{2\theta}{2}\cos\alpha} = -\tan\frac{2\theta}{2}\tan\alpha\pm\frac{\cos\sqrt{w_\mu^2+2\left(\frac{2\theta}{2}-\alpha\right)^2}}{\cos\frac{2\theta}{2}\cos\alpha} \quad \text{(S11)}$$

The two solutions correspond to the upper and lower peaks. Considering the upper peak only, the χ angle for the HWHM on either side of the peak at π/2 is given (a) below π/2: taking the arcsine of $\sin\chi_+^{(00l)}$ and (b) above π/2, π minus the value in (a).

S5.2. (hk0)

For (hk0), $\mu = \frac{\pi}{2}$, and the angular term P_θ is maximum (1) when

$$\frac{\sin\frac{2\theta}{2}\sin\alpha+\cos\frac{2\theta}{2}\sin\chi\cos\alpha}{\sqrt{\left(\cos\frac{2\theta}{2}\cos\chi\right)^2+\left(\sin\frac{2\theta}{2}\cos\alpha-\cos\frac{2\theta}{2}\sin\chi\sin\alpha\right)^2}} = 0 \quad \text{(S12)}$$

i.e.

$$\sin\chi = -\frac{\sin\frac{2\theta}{2}\sin\alpha}{\cos\frac{2\theta}{2}\cos\alpha} = -\tan\frac{2\theta}{2}\tan\alpha \quad \text{(S13)}$$

which corresponds to points below 0 and above 180°.

For the FWHM, we need to solve the equation below for χ

$$\frac{\sin\frac{2\theta}{2}\sin\alpha+\cos\frac{2\theta}{2}\sin\chi\cos\alpha}{\sqrt{\left(\cos\frac{2\theta}{2}\cos\chi\right)^2+\left(\sin\frac{2\theta}{2}\cos\alpha-\cos\frac{2\theta}{2}\sin\chi\sin\alpha\right)^2}} = \tan w_\mu \quad \text{(S14)}$$

which can be written as a quadratic equation in $\sin\chi$. Solving, we get

$$\sin\chi_\pm^{(hk0)} = \frac{-\sin\frac{2\theta}{2}\sin\alpha\pm\cos w_\mu}{\cos\frac{2\theta}{2}\cos\alpha} = -\tan\frac{2\theta}{2}\tan\alpha\pm\frac{\cos w_\mu}{\cos\frac{2\theta}{2}\cos\alpha} \quad \text{(S15)}$$

Here, the ± signs have the following meaning: the arcsine of $\sin\chi_+^{(hk0)}$ gives the HWHM to the right of the peak near 0°, as also the HWHM to the *left* of the peak near 180°. Conversely, the arcsine of $\sin\chi_-^{(hk0)}$ is negative. When added to the peak near 360° (or 0°), the arcsine gives the angle at which HWHM is reached to its left, and when subtracted from the peak near 180°, gives the angle at which HWHM is reached to its *right*.

S5.3. (hkl) or (0kl)

For the (013) and similar (0kl) cases, μ is between 0 and $\frac{\pi}{2}$. To find the maxima, we solve for where the rings intersect the Ewald sphere, corresponding to the angular maxima of P_θ, i.e. where:

$$\tan^{-1}\left(\frac{\sin\frac{2\theta}{2}\sin\alpha+\cos\frac{2\theta}{2}\sin\chi\cos\alpha}{\sqrt{\left(\cos\frac{2\theta}{2}\cos\chi\right)^2+\left(\sin\frac{2\theta}{2}\cos\alpha-\cos\frac{2\theta}{2}\sin\chi\sin\alpha\right)^2}}\right)=\frac{\pi}{2}-\mu \qquad \text{(S16)}$$

which is equivalent to

$$\tan^2\mu\left(\sin\frac{2\theta}{2}\sin\alpha+\cos\frac{2\theta}{2}\sin\chi\cos\alpha\right)^2=\left(\cos\frac{2\theta}{2}\cos\chi\right)^2+\left(\sin\frac{2\theta}{2}\cos\alpha-\cos\frac{2\theta}{2}\sin\chi\sin\alpha\right)^2 \qquad \text{(S17)}$$

The equation has the following solutions in χ:

$$\sin\chi_\pm^\mu=\frac{-\sin\frac{2\theta}{2}\sin\alpha\pm\cos\mu}{\cos\frac{2\theta}{2}\cos\alpha}=-\tan\frac{2\theta}{2}\tan\alpha\pm\frac{\cos\mu}{\cos\frac{2\theta}{2}\cos\alpha} \qquad \text{(S18)}$$

Using the values for the (013) chitin peak, with $\mu\sim 10.3°$, it can be confirmed that χ_+ corresponds to the upper branch (1st quadrant) split peak, whilst $2\pi+\chi_-$ corresponds to the 4th quadrant split peak (since the arcsine returns angles between $-\frac{\pi}{2}$ and $\frac{\pi}{2}$. The other two split peaks are at $\pi-\chi_+$ (2nd quadrant) and $\pi+\chi_-$ (3rd quadrant) respectively.

The split peak range for the upper and lower peaks is from $\alpha=0$ to $\alpha=\alpha_U^c,\alpha_L^c$ where

$$1=-\tan\frac{2\theta}{2}\tan\alpha_U^c+\frac{\cos\mu}{\cos\frac{2\theta}{2}\cos\alpha_U^c} \qquad \text{(S18)}$$

and

$$-1=-\tan\frac{2\theta}{2}\tan\alpha_U^c-\frac{\cos\mu}{\cos\frac{2\theta}{2}\cos\alpha_U^c} \qquad \text{(S19)}$$

The first equation can be solved by setting $\tan\alpha_U^c=x$, leading to

$$1+x\tan\frac{2\theta}{2}=r\sqrt{x^2+1} \text{ where } r=\frac{\cos\mu}{\cos\frac{2\theta}{2}} \qquad \text{(S20)}$$

which leads to

$$1+x^2\tan^2\frac{2\theta}{2}+2x\tan\frac{2\theta}{2}=r^2(x^2+1)$$

Or

$$x^2\left(\tan^2\frac{2\theta}{2}-r^2\right)+2x\tan\frac{2\theta}{2}+(1+r^2)=0 \qquad \text{(S21)}$$

The solution is

$$\tan\alpha_U^c=\frac{-\sin\frac{2\theta}{2}\cos\frac{2\theta}{2}\pm\cos\mu\sin\mu}{\sin^2\frac{2\theta}{2}-\cos^2\mu} \qquad \text{(S22)}$$

Using the SAXS limit $\frac{2\theta}{2}\to 0$ to select the correct sign (since $\alpha_U^c=\mu$ in this case), we have, for the upper 1st quadrant, the critical angle

$$\tan\alpha_U^c=\frac{\cos\mu\sin\mu+\sin\frac{2\theta}{2}\cos\frac{2\theta}{2}}{\cos^2\mu-\sin^2\frac{2\theta}{2}} \qquad \text{(S23)}$$

This is the same angle as for the 2nd quadrant intersection, by symmetry.

In a similar manner, the second equation can be solved as:

$$-1 + x\tan\frac{2\theta}{2} = -r\sqrt{x^2+1} \qquad \text{(S24)}$$

Which has the solutions:

$$\tan\alpha_L^c = \frac{\sin\frac{2\theta}{2}\cos\frac{2\theta}{2} \pm \cos\mu\sin\mu}{\sin^2\frac{2\theta}{2} - \cos^2\mu} \qquad \text{(S25)}$$

As above, the solution for a positive α tilt is obtained with the -ve sign in the branch, hence

$$\tan\alpha_L^c = \frac{\cos\mu\sin\mu - \sin\frac{2\theta}{2}\cos\frac{2\theta}{2}}{\cos^2\mu - \sin^2\frac{2\theta}{2}} \qquad \text{(S26)}$$

This angle will be smaller than α_L^c, as expected, since the lower ring goes out of intersection condition faster than the upper one.

We have three ranges for the intensity ratios:

1. $\alpha < \alpha_L^c$: The peak intensity ratio is 1; both peaks are split
2. $\alpha_L^c \leq \alpha < \alpha_U^c$: The lower peak merges into a single peak and reduces in intensity, the upper peaks are split with a constant maximum
3. $\alpha \geq \alpha_L^c$: Both split peaks have merged and reduce in intensity.

From visual inspection of the (013) peaks it should be feasible to identify which stage (1-3) we are in, following which the intensity ratios can be calculated, by substituting the peak angles from (S18) into the Θ (Eq. (s4)) and P_θ functions.

For the FWHM, a similar equation is obtained, where:

$$\frac{\sin\frac{2\theta}{2}\sin\alpha + \cos\frac{2\theta}{2}\sin\chi\cos\alpha}{\sqrt{\left(\cos\frac{2\theta}{2}\cos\chi\right)^2 + \left(\sin\frac{2\theta}{2}\cos\alpha - \cos\frac{2\theta}{2}\sin\chi\sin\alpha\right)^2}} = \tan\left(\frac{\pi}{2} - \mu \pm w_\mu\right) \qquad \text{(S27)}$$

which has solutions of the form (all possible $\pm$ combinations)

$$\sin\chi_\pm^{\mu;hwhm} = \frac{-\sin\frac{2\theta}{2}\sin\alpha \pm \cos(\mu \mp w_\mu)}{\cos\frac{2\theta}{2}\cos\alpha} = -\tan\frac{2\theta}{2}\tan\alpha \pm \frac{\cos(\mu \mp w_\mu)}{\cos\frac{2\theta}{2}\cos\alpha} \qquad \text{(S28)}$$

This will not necessarily have solutions for all μ, α: e.g. for small μ, α only one HWHM value is found for each of the 4 (2x2 split) peaks. This is due to the overlapping of the split peaks both in the upper and lower quadrants, and the effect becomes stronger for larger w_μ. It can be seen numerically that for the HWHM on the left-hand side (in a $I(\chi)$ plot) is obtained by finding

$$\sin\chi_1^{\mu;hwhm} = -\tan\frac{2\theta}{2}\tan\alpha + \frac{\cos(\mu + w_\mu)}{\cos\frac{2\theta}{2}\cos\alpha} \qquad \text{(S29)}$$

and taking the difference between $\chi_+^\mu - \chi_1^{\mu;hwhm}$ (plotted in Fig 3E-iii, main text).

We note that some of the simplifications which have led to the above expressions are as far as we can tell a fortuitous choice of Lorentzians rather than Gaussians in P_θ.

S5.4. Limiting expressions for azimuthal intensity expressions for (*00l*) and (*hk0*):

Using Lorentzians instead of Gaussians for the angular intensity profile is a basic difference here from our previous papers [3–5]. However, using the equivalent peak profile for a Gaussian instead of a Lorentzian close to the peak position, it is possible to show a match for the (00l) and (hk0) cases considered there. Since we are only considering the scattering vector **q**, in this case we write (Q_x, Q_y, Q_z) as equivalent to (q_x, q_y, q_z), when used in the δ and Θ expressions.

In the following, the reciprocal space vector **Q** is set equal to the scattering vector $\boldsymbol{q} = \boldsymbol{k}_2 - \boldsymbol{k}_1 = \frac{4\pi}{\lambda}\sin\frac{2\theta}{2}$, which can be expressed in component form as in Table S1, last row.

Starting from

$$\Theta(\boldsymbol{q},0) = \tan^{-1}\left(\frac{q\sin\frac{2\theta}{2}\sin\alpha + q\cos\frac{2\theta}{2}\sin\chi\cos\alpha}{\sqrt{\left(q\cos\frac{2\theta}{2}\cos\chi\right)^2 + \left(q\sin\frac{2\theta}{2}\cos\alpha - q\cos\frac{2\theta}{2}\sin\chi\sin\alpha\right)^2}}\right) \quad \text{(S30)}$$

And setting α=0 (no tilt), we have

$$\Theta(\boldsymbol{q},0) = \tan^{-1}\left(\frac{q\cos\frac{2\theta}{2}\sin\chi}{\sqrt{\left(q\cos\frac{2\theta}{2}\cos\chi\right)^2 + \left(q\sin\frac{2\theta}{2}\right)^2}}\right) \quad \text{(S31)}$$

The angular intensity expression is therefore

$$\delta_\mu(\boldsymbol{q}) = P_\theta\left(\tan^{-1}\left(\frac{q\cos\frac{2\theta}{2}\sin\chi}{\sqrt{\left(q\cos\frac{2\theta}{2}\cos\chi\right)^2 + \left(q\sin\frac{2\theta}{2}\right)^2}}\right); \mu, w_\mu\right) \quad \text{(S32)}$$

S5.5. (110)

For the (110) reflection, we have

$$P_\theta\big(\Theta(\boldsymbol{q}); \mu, w_\mu\big) = \frac{1}{\pi}\frac{w_\mu}{\left(\Theta(\boldsymbol{q}) - \left(\frac{\pi}{2} - \mu_{(110)}\right)\right)^2 + w_\mu^2} \quad \text{(S33)}$$

Since $\mu_{(110)}=\pi/2$, we have

$$P_\theta\big(\Theta(\boldsymbol{q}); \mu, w_\mu\big) = \frac{1}{\pi}\frac{w_\mu}{\Theta(\boldsymbol{q})^2 + w_\mu^2} \quad \text{(S34)}$$

The above expression will be maximum when $\Theta(\boldsymbol{q}) = 0$ which occurs at χ=0,π as expected for (110). Since the argument of $\tan^{-1}$ is small, we can approximate $\tan^{-1}x \to x$ as $x \to 0$, hence

$$P_\theta\big(\Theta(\boldsymbol{q}); \mu, w_\mu\big) = \frac{1}{\pi}\frac{w_\mu}{\left(\frac{q\cos\frac{2\theta}{2}\sin\chi}{\sqrt{\left(q\cos\frac{2\theta}{2}\cos\chi\right)^2 + \left(q\sin\frac{2\theta}{2}\right)^2}}\right)^2 + w_\mu^2} \quad \text{(S34)}$$

The original expression used in Eqn. (2) in [3] features a Gaussian peak function rather than Lorentzian. If we have a peak shaped profile centred at 0 with a width w, then the Gaussian function $\exp\left(-\frac{(x)^2}{2w^2}\right)$

and the Lorentzian function $\frac{1}{1+\left(\frac{x}{w}\right)^2}$ are both peak profiles which can be used to fit it. Making the switch here, we have

$$P_\theta\big(\Theta(\boldsymbol{q});\mu,w_\mu\big) \cong \exp\left(-\frac{1}{2w_\mu^2}\frac{\left(q\cos\frac{2\theta}{2}\sin\chi\right)^2}{\left(q\cos\frac{2\theta}{2}\cos\chi\right)^2+\left(q\sin\frac{2\theta}{2}\right)^2}\right) \text{(S35)}$$

If most of the intensity is concentrated around the χ=0,π band, the cosine term in the denominator is ~1. Since for the small scattering angles considered here, $q \sim q\cos\frac{2\theta}{2} \gg q\sin\frac{2\theta}{2}$, we have

$$P_\theta\big(\Theta(\boldsymbol{q});\mu,w_\mu\big) \cong \exp\left(-\frac{1}{2}\frac{q_y^2}{\left(w_\mu q_R\right)^2}\right) \quad \text{(S36)}$$

In [3], the fibre was oriented along the *x*-axis, while here it is along the *y*-axis. The expression therefore now matches Eqn. (2) in [3], which is

$$\delta(q_x;a_x) \cong \exp\left(-\frac{1}{2}\left(\frac{q_x}{a_x}\right)^2\right) \quad \text{(S37)}$$

S5.6. (002)

For the (002) reflection, we have

$$P_\theta\big(\Theta(\boldsymbol{q});\mu,w_\mu\big) = \frac{1}{\pi}\frac{w_\mu}{\left(\Theta(\boldsymbol{q})-\left(\frac{\pi}{2}-\mu_{(110)}\right)\right)^2+w_\mu^2} \quad \text{(S38)}$$

Becomes $\mu_{(002)}$=0, we have

$$P_\theta\big(\Theta(\boldsymbol{q});\mu,w_\mu\big) = \frac{1}{\pi}\frac{w_\mu}{\left(\Theta(\boldsymbol{q})-\frac{\pi}{2}\right)^2+w_\mu^2} \quad \text{(S39)}$$

The above expression will be maximum when $\left|\Theta(\boldsymbol{q})-\frac{\pi}{2}\right|$ is minimum, i.e. when $\Theta(\boldsymbol{q},0) = \tan^{-1}\left(\frac{q\cos\frac{2\theta}{2}\sin\chi}{\sqrt{\left(q\cos\frac{2\theta}{2}\cos\chi\right)^2+\left(q\sin\frac{2\theta}{2}\right)^2}}\right) \to \frac{\pi}{2}$. For $\frac{q^2}{2q_E} \ll q_R$ that occurs when χ→π/2, as expected, and the intensity is effectively nonzero within a width of w_μ. Using $\tan^{-1}x = sgn(x)\frac{\pi}{2} - \tan^{-1}\frac{1}{x}$ we can write the above expression as:

$$sgn\left(\frac{q\cos\frac{2\theta}{2}\sin\chi}{\sqrt{\left(q\cos\frac{2\theta}{2}\cos\chi\right)^2+\left(q\sin\frac{2\theta}{2}\right)^2}}\right)\frac{\pi}{2} - \tan^{-1}\left(\frac{\sqrt{\left(q\cos\frac{2\theta}{2}\cos\chi\right)^2+\left(q\sin\frac{2\theta}{2}\right)^2}}{q\cos\frac{2\theta}{2}\sin\chi}\right) \text{(S40)}$$

Consider the upper quadrant for simplicity; the sign term is positive, and we have

$$\frac{\pi}{2} - \tan^{-1}\left(\frac{\sqrt{\left(q\cos\frac{2\theta}{2}\cos\chi\right)^2+\left(q\sin\frac{2\theta}{2}\right)^2}}{q\cos\frac{2\theta}{2}\sin\chi}\right) \quad \text{(S41)}$$

where the term in parentheses is small as it approaches χ→π/2. Since $\tan^{-1}x \to x$ it simplifies to

$$\frac{\pi}{2} - \frac{\sqrt{\left(q\cos\frac{2\theta}{2}\cos\chi\right)^2+\left(q\sin\frac{2\theta}{2}\right)^2}}{q\cos\frac{2\theta}{2}\sin\chi} \quad \text{(S42)}$$

The expression is therefore

$$P_\theta\big(\Theta(\boldsymbol{q});\mu,w_\mu\big)\approx\frac{1}{\pi}\frac{w_\mu}{\left(\frac{\sqrt{\left(q\cos\frac{2\theta}{2}\cos\chi\right)^2+\left(q\sin\frac{2\theta}{2}\right)^2}}{q\cos\frac{2\theta}{2}\sin\chi}\right)^2+w_\mu^2}\qquad\text{(S43)}$$

Making the same exponential/Lorentzian peak switch and $q\sim q\cos\frac{2\theta}{2}$ we have

$$P_\theta\big(\Theta(\boldsymbol{q});\mu,w_\mu\big)\cong\exp\left(-\frac{1}{2w_\mu^2}\frac{\left(q\cos\frac{2\theta}{2}\cos\chi\right)^2+\left(q\sin\frac{2\theta}{2}\right)^2}{\left(q\cos\frac{2\theta}{2}\sin\chi\right)^2}\right)=\exp\left(-\frac{1}{2w_\mu^2}\frac{q_x^2+q_z^2}{q_y^2}\right)\qquad\text{(S44)}$$

Near the peak at $\chi=\pi/2$, we have $|q|\sim|q_y|$ hence

$$P_\theta\big(\Theta(\boldsymbol{q});\mu,w_\mu\big)\cong\exp\left(-\frac{1}{2w_\mu^2}\frac{q_x^2+q_z^2}{q^2}\right)\qquad\text{(S45)}$$

Since the intensity is evaluated for $q=q_{(002)}$, the expression can be written as

$$P_\theta\big(\Theta(\boldsymbol{q});\mu,w_\mu\big)\cong\exp\left(-\frac{1}{2}\frac{q_x^2}{w_\mu^2q^2}\right)\exp\left(-\frac{1}{2}\frac{q_z^2}{w_\mu^2q^2}\right)\qquad\text{(S46)}$$

In [4], our model had expressed the above term in an asymmetric manner between q_x and q_z; taking the Dirac δ-function limit ($\delta(x)=\lim_{a\to0}\frac{1}{\sqrt{\pi a}}\exp(-ax^2)$) for the q_z term while retaining the exponential for the q_x term to get a term of the form (Equation (1) in [4]):

$$\exp\left(-\frac{1}{2}\frac{q_x^2}{\Delta q_{(002)}^2}\right)\delta(q_z)\qquad\text{(S47)}$$

S6. Supplementary References:

1. Stribeck N. (2009). Acta Cryst. A65, 46–7.
2. Polanyi M. (1921). Z. Physik 7, 149–80.
3. Zhang, Y., Paris, O., Terrill, N. J. & Gupta, H. S. (2016). Sci. Rep. 6, 26249.
4. Zhang, Y., De Falco, P., Wang, Y., Barbieri, E., Paris, O., Terrill, N. J. et al. (2017). Nanoscale 9, 11249–60.
5. Zhang, Y., Garrevoet, J., Wang, Y., Roeh, J. T., Terrill, N. J., Falkenberg, G. et al. (2020). ACS Nano 14.
